# The interplay among space, environment, and gene flow drives genetic differentiation in endemic Baja California *Agave sobria* subspecies

**DOI:** 10.1002/ajb2.70062

**Published:** 2025-07-02

**Authors:** Anastasia Klimova, Jesús Gutiérrez Rivera, Oscar E. Juárez, Alfredo Ortega Rubio, Luis E. Eguiarte

**Affiliations:** ^1^ Centro de Investigaciones Biológicas del Noroeste S.C. La Paz Baja California Sur Mexico; ^2^ Departamento de Ecología Evolutiva, Instituto de Ecología Universidad Nacional Autónoma de México, Ciudad Universitaria, Circuito Exterior s/n Annex to the Botanical Garden Mexico City 04510 Mexico

**Keywords:** Agavoideae, isolation by distance, isolation by environment, phylogeography, spatial population genetics

## Abstract

**Premise:**

Research on neutral and adaptive processes that lead to the divergence of species and populations is a crucial component in evolutionary and conservation genetics. *Agave sobria* is an endemic group of subspecies scattered on canyons along a latitudinal gradient and distinct environments of the Baja California Peninsula, Mexico. *Agave sobria* represents a unique opportunity to study the process of genetic differentiation in a highly heterogeneous environment.

**Methods:**

Using genotyping‐by‐sequencing, we genotyped 8453 single‐nucleotide polymorphisms (SNPs) in all *A. sobria* subspecies, including 19 *A. sobria* and three closely related *A. cerulata* ssp. *subcerulata* populations. We assessed neutral genetic structure and diversity at both the interspecific and intraspecific levels, evaluated the amount and direction of gene flow, and identified putatively adaptive SNPs.

**Results:**

We found low support for the currently recognized subspecies. We detected neutral (i.e., isolation by distance) and adaptive divergence linked to eco‐geographic characteristics of the habitat. High genetic connectivity indicated that gene flow between central and northern populations may have homogenizing effects preventing population differentiation. For the southernmost *A. sobria* ssp. *frailensis*, temperature and geographic isolation appear to be the main drivers of adaptive differentiation, with outlier SNPs located in coding regions involved in response to abiotic stress and immunology.

**Conclusions:**

In *A. sobria*, environmental isolation and geographic gradients affect the genetic structure, creating opportunities for local adaptation. Our results emphasize the importance of including neutral and adaptive perspectives, the combination of which allows a better understanding of the complexity of the processes that lead to population differentiation.

Determining genetic structure and understanding its underlying causes is essential in evolutionary and conservation genetic studies (Höglund, 2009; Allendorf et al., 2012). Environmental differences and geographic distance are important elements that determine the genetic structure of natural plant populations (Loveless and Hamrick, 1984; Holderegger et al., 2010). The former is related to adaptability to biotic and abiotic pressures. Thus, in a highly heterogeneous landscape where plant populations are subject to habitat‐specific selective pressures, divergent selection will reduce gene flow and promote divergence via genetic drift and local adaptation (Nosil et al., 2009; Orsini et al., 2013). Selection will determine the genetic diversity of genes associated with adaptation and of neutral regions associated with fitness‐related genetic background (Walsh and Lynch, 2018; Chung et al., 2023). The synergy between genetic drift and variation‐reducing selection will make the distribution of genetic variation among populations uneven and the genetic differentiation stronger than expected on the basis of geographic separation alone (Loveless and Hamrick, 1984; Orsini et al., 2013). Geographic distance, however, is relevant for genetic structure through the interaction between genetic drift that promotes population divergence and gene flow that homogenizes populations (Wright, 1943, 1946; Clegg and Phillimore, 2010). The tug of war between genetic drift and gene flow results in the classic pattern of isolation by distance (IBD; Sokal, 1979; Rousset, 1997).

Ecological factors like precipitation and temperature regimes can be critical in defining the genetic structure of populations, especially in plants that grow in harsh and water‐limited environments (Loveless and Hamrick, 1984; Slatkin, 1987; Mosca et al., 2013). In heterogeneous landscapes, natural plant populations can be subject to habitat‐specific selective pressures (Kawecki and Ebert, 2004; Lascoux et al., 2016). Over time, if the selective pressure is relatively high and the gene flow is restricted, genetic differences among populations may lead to local adaptation and, eventually, to speciation (Hedrick et al., 1976; Hedrick, 1986; Leimu and Fischer, 2008). Thus, incorporating environmental information into a population genetics framework may be helpful in determining the factors that modulate the strength and interactions among major evolutionary forces (i.e., selection, genetic drift, mutation, and gene flow), ultimately defining and understanding processes leading to speciation (Manel and Segelbacher, 2009).

The Sonoran Desert is vast and relatively young (ca. 8–15 million years old; Axelrod, 1979; Van Devender, 1990; McAuliffe and Van Devender, 1998). A significant portion of the Sonoran Desert is located in the Baja California Peninsula (BCP), Mexico, one of the longest and most isolated peninsulas in the world (Grismer, 2000; González‐Abraham et al., 2010). The complex geomorphology, the marked differences in climatic conditions along the BCP, and the longtime isolation have resulted in a highly heterogeneous landscape and have promoted the existence of unique plant communities (Gentry, 1978; Riemann and Ezcurra, 2005). This remarkable floral gradient includes coastal vegetation, high‐altitude coniferous forest, and desert scrubs in <100 km of the east‐west transects (Gentry, 1978; Riemann and Ezcurra, 2005; Riemann and Exequiel, 2007). The sierras that stretch along the peninsula form “sky island” refuges, where numerous Pleistocene relicts are found (Axelrod, 1979; Moran, 1977, 1983). However, >75% of the peninsula is arid, and the vegetation is dominated by plant communities adapted to environmental extremes, such as columnar cacti, agaves, yuccas, and several shrub species (Gentry, 1978; Riemann and Ezcurra, 2005; Riemann and Exequiel, 2007; González‐Abraham et al., 2010).

*Agave* L. (Asparagaceae) is a species‐rich genus with >200 taxa that originated in Mexico (Gentry, 1982; Eguiarte et al., 2021). The crassulacean acid metabolism used by agaves granted them a tremendous advantage for flourishing in hot and dry climates (Eguiarte et al., 2021). However, being a hallmark of arid and semiarid landscapes, agaves can be found growing from coastal dunes at sea level up to oak‐pine and cloud forests at >2500 m a.s.l. (Gentry, 1982; Eguiarte et al., 2021). *Agave* species are characterized by relatively low within‐species genetic structure, explained by the gene flow over large geographic distances promoted by their primary pollinators, particularly nectarivorous bats (e.g., *Leptonycteris* spp.; Eguiarte et al., 2013; Flores‐Torres and Galindo‐Escamilla, 2017; Gómez‐Ruiz and Lacher, 2019). However, dispersal potential may vary across fragmented landscapes due to geographic and/or environmental factors such as mountain ranges, low‐elevation deserts, temperature, and precipitation variations (Gómez‐Ruiz and Lacher, 2019; Cruzan and Hendrickson, 2020).

Indeed, recent studies have shown complex patterns of genetic connectivity related to historical demography, Quaternary climate fluctuations, human use, and geography in a wide range of agave species, including *A. angustifolia* Haw. (Klimova et al., 2022), *A. potatorum* Zucc. (Ruiz‐Mondragón et al., 2023), *A. marmorata* Roezl. (Ruiz‐Mondragón et al., 2024), *A. aurea* Brandegee (Klimova et al., 2024), *A. lechuguilla* Torr. (Scheinvar et al., 2016), and *A. parryi* Engelm. (Lindsay et al., 2018). However, most of these studies in agaves did not explicitly consider the potential impacts of environmental factors on adaptive genetic variation. An “adaptive” perspective is desirable, given that the critical questions of how and where gene flow is constrained are tightly linked to the fitness of individuals in their environment (Lenormand, 2002, 2012). Therefore, elucidating the geographic and environmental determinants of population structure and local adaptation is a crucial step that will enable informed responses to essential questions in desert plant evolution and conservation.

Agaves are well known for high levels of endemism and many micro‐endemic species (Gentry, 1982; Eguiarte et al., 2021; Alducin‐Martínez et al., 2022). For instance, among 23 known agave taxa on the BCP, 22 are endemic (Webb and Starr, 2015). Within this variability of forms and shapes, *A. sobria* Brandegee represents an interesting case, in part because it is a group of three currently recognized subspecies that grows on rocky slopes of the southern half of the BCP (Gentry, 1982; Webb and Starr, 2015). Apparently, the three subspecies combine sexual and asexual reproduction, and all of them have extremely high morphological variability (Webb and Starr, 2015). *Agave sobria* ssp. *sobria* is the most common subspecies, and it is scattered but common on Sierra La Giganta slopes (Figure 1). *Agave sobria* ssp. *frailensis* has restricted distribution and grows on granite hillslopes at only a few localities along the Gulf of California, between Cabo Frailes and Punta Los Mangles (Figure 1). Finally, *A. sobria* ssp. *roseana* occurs on the coasts north and east of La Paz city and islands just offshore, particularly Isla Espiritu Santo (Webb and Starr, 2015). Interestingly, in the most northerly forms of *A. sobria* in the mountains between La Purísima and Mulegé, clonal propagation predominates, and these populations have apparent affinities to another agave species, *A. cerulata* Trel. (Gentry, 1978; Webb and Starr, 2015). All the subspecies prefer shadier canyons, and do not form dense colonies, whereas plants growing at a lower elevation at the mountain base are usually smaller and in poorer physical condition due to drought (Gentry, 1978).

**Figure 1:**
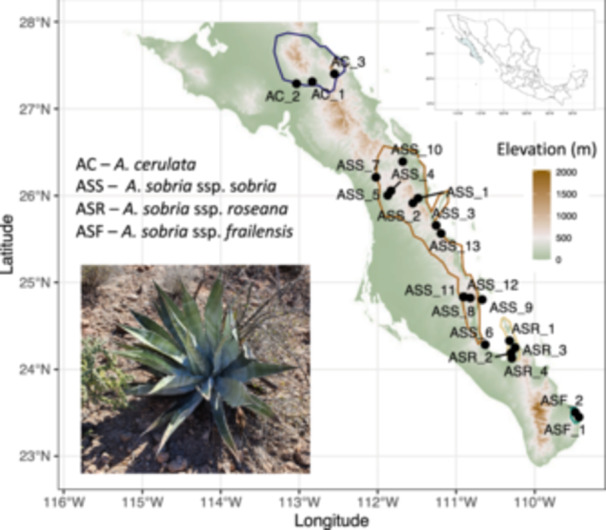
Map of the southern Baja California Peninsula, Mexico, with a background representing the topography of the area and black dots representing 22 sample site locations of *Agave sobria* and *A. cerulata* ssp. *subcerulata*. Colored lines around sampling sites represent the potential distribution of each sampled subspecies: green = *A. sobria* ssp. *frailensis*, light brown = *A. sobria* ssp. *roseana*, dark brown = *A. sobria* ssp. *sobria*, and dark blue = *A. cerulata* ssp. *subcerulata* (modified from Webb and Starr, 2015). Inset is a picture of *A. sobria* ssp. *sobria* collected in Sierra La Giganta.

The geographic distribution of *A. sobria* spans a latitudinal gradient, which, coupled with clonal reproduction, may suggest “neutral” genetic divergence expressed as isolation by distance. However, the latitudinal gradient is also related to the environmental differences between the southernmost tip of the peninsula and the drier northern portion of the species' distribution range (Gentry, 1978; Riemann and Exequiel, 2007; De la Luz et al., 2008; Webb and Starr, 2015). This ecological difference opens the possibility of adaptive differentiation among populations (subspecies) and even incipient speciation. Documenting neutral and adaptive genetic structure is important not only in a broad sense of understanding forces that promote divergence and speciation but also in a narrow sense of establishing conservation and management units (Mable, 2018; Chung et al., 2023). Determining the genetic basis of local adaptation to temperature and precipitation is particularly interesting, as it can be considered a primary objective of conservation biology since it could help forecast how desert species will react to ongoing climate change (Hoffmann et al., 2021; Mohanta et al., 2024).

The goals of this study were to (1) clarify the relationships within and among the *A. sobria* subspecies using genomic data; (2) evaluate patterns of the genetic diversity distribution and unravel their underlying factors; and (3) assess the potential role of spatial distribution and environmental factors in shaping neutral and adaptive genetic structure in *A. sobria*. To accomplish these goals, we applied phylogenetic analysis, landscape genomics, population differentiation, and environmental association approaches to samples of all the *A. sobria* subspecies spanning the complete distribution range of this species.

## MATERIALS AND METHODS

### Sample collection

In 2023, we sampled 19 *A. sobria* and three *A. cerulata* populations in the BCP (Figure 1; Appendix S1). All three currently recognized subspecies of *A. sobria* (*A. sobria* ssp. *sobria*, *A. sobria* ssp. *roseana*, *A. sobria* ssp. *frailensis*) were included in the sampling scheme. Only the southernmost subspecies of *A. cerulata* (i.e., *A. cerulata* ssp. *subcerulata*) was included to be used as the outgroup for the *A. sobria* subspecies (the other subspecies of *A. cerulata* are geographically more distant to *A. sobria*, see Gentry (1982) and Navarro‐Quezada et al. (2003)). From each sampling locality, we collected three to six plants. To avoid sampling clonal ramets, we sampled individuals at least 5 meters apart or further from each other. Leaves from individual specimens were kept in paper bags at room temperature, away from heat and direct sunlight. At the laboratory in the Centro de Investigaciones Biológicas del Noroeste S.C., La Paz, Baja California Sur, Mexico, samples were kept at −20°C until DNA extraction. The final number of the collected samples was 69 for *A. sobria* and nine for *A. cerulata*. Details on geographic coordinates and the number of specimens collected at each locality can be found in Appendix S1. We also included nine samples of *A. aurea* from Klimova et al. (2024); these samples were used in phylogenetic analysis and acted as a more distant outgroup to *A. sobria* and *A. cerulata* (Webb and Starr, 2015).

### Molecular analysis and SNP selection

For DNA extraction, three or four individuals were used from each sampling site (Appendix S1). Total DNA was extracted from leaf tissue using a modified CTAB protocol (Doyle and Doyle, 1987); the step‐by‐step protocol can be found in Klimova et al. (2022). DNA quality and quantity were evaluated by electrophoresis in 1% agarose gel and a Qubit 3.0 fluorometer (Qubit dsDNA broad‐range kit). High‐quality DNA was sent to the University of Wisconsin Biotechnology Center for RADseq library preparation (Elshire et al., 2011; Andrews et al., 2016), and 150‐bp paired‐end sequencing using the Illumina NovaSeq platform (Illumina, San Diego, California, USA). A combination of methylation‐sensitive restriction enzymes (PstI/MspI) successfully used in previous studies of agave (Klimova et al., 2022; Ruiz‐Mondragón et al., 2024) was used for DNA fragmentation and library preparation.

Raw sequences were filtered using the fastp (Chen et al., 2018) by removing adapters, short (<55 bp), low‐quality, and low‐complexity (highly repetitive) reads. We also trimmed poly G and poly X tails and removed sequences with over five N. Filtered reads were demultiplexed using the process_radtags function in STACKS version 1.41 (Catchen et al., 2013; Rochette et al., 2019) and mapped to the *A. tequilana* transcriptome (GAHU00000000.1; Gross et al., 2013) using BWA version 0.7.13 (Li and Durbin, 2009). The SAM files were converted to BAM format, sorted by coordinates, and indexed using SAMtools (Danecek et al., 2021). Single‐nucleotide polymorphisms (SNPs) were called using bcftools (Danecek et al., 2021). The posterior variant filtering was performed using VCFtools version 0.1.15 (Danecek et al., 2011). Only loci with a mean depth of >10 and <200 and a maximum of two alleles with no insertions or deletions were kept. Additionally, we set a minor allele count at six to reduce the possibility of including singletons or removing rare alleles (Linck and Battey, 2019). We excluded sites based on the proportion of missing data, keeping sites with no more than 10% missing data (max‐missing 0.9).

We then filtered out the variants that significantly deviated from Hardy‐Weinberg equilibrium (*p* ≤ 0.05 after multiple test corrections). We also estimated the correlation between each pair of loci (*r* ^2^). Then, to ameliorate the confounding effects of linkage disequilibrium (LD), we eliminated markers with *r* ^2^ > 0.2 as implemented in PLINK (‐indep‐pairwise 50 5 0.2; Purcell et al., 2007). We used function “–thin” from VCFtools to reduce linkage among loci further and “mlg” function implemented in the R package poppr to determine the genotype's uniqueness (Kamvar et al., 2014).

Three final data sets were created: (1) phylogenetic and connectivity (ABBA BABA test) data sets including *A. sobria*, *A. cerulata* ssp. *subcerulata*, and *A. aurea*; (2) genome scan for outlier loci, including only *A. sobria*; and (3) the rest of the analyses, which included *A. sobria* and *A. cerulata* ssp. *subcerulata*. The details of the data analyses can be found in Appendix S2 in the form of a flow chart, including the programs used for neutral and adaptive approaches.

### Phylogenetic analysis

To determine phylogenetic relationships among the morphologically described subspecies and to delimit the geographic boundaries of each group, we utilized SVDquartets analysis (singular value decomposition, SVD, scores), a phylogenetic method designed by Chifman and Kubatko (2015). SVDquartets are useful for assessing the species‐tree topology based on SNP data under the multi‐species‐coalescent model (Chou et al., 2015) by calculating the SVD score (Chifman and Kubatko, 2015) for each possible four‐species (quartet) topology in the data set, and by optimizing the overall tree topology to maximize these scores. The SVD score itself is calculated based on a matrix of the genotype probabilities in a given quartet and on the dimensionality of this matrix (Chifman and Kubatko, 2015). We examined all possible quartets among our samples, with individuals clustered at the sampling site level, and estimated branch support using 500 bootstrap replicates in PAUP* version 4.0a150 (Swofford, 2003). We used nine samples of *A. aurea* as an outgroup.

### Population genetic structure

Genetic differentiation between subspecies and among individuals was explored using principal component analysis (PCA), individual genetic clustering, and distance‐based relationships. First, we visualized genetic relationships among samples using PCA, an efficient nonmodel method for assessing population structure in high‐dimensional data sets (Patterson et al., 2006), using the R package SNPrelate (Zheng et al., 2012). We also utilized a clustering approach based on a maximum likelihood estimation of individual ancestries as implemented in ADMIXTURE version 1.23 (Alexander et al., 2009). Clusters in ADMIXTURE were set from 1 to 10 (*K*), with 20 replicates for each *K* value. The support for different *K* values was assessed according to the replicates' likelihood distribution (i.e., lowest cross‐validation error). To further assess and visualize the genetic relationships among samples, we constructed a distance tree using the R package poppr (Kamvar et al., 2014). The distance tree was based on the neighbor‐joining (NJ) algorithm, with 1000 bootstrap replicates to assess branch support. In addition, we estimated pairwise *F*_ST_ between subspecies, among sampling sites, and among geographic regions using the StAMPP package (Pembleton et al., 2013).

### Spatial genetic structure and migration

To evaluate the relevance of geography in structuring agave populations, we used SpaceMix (Bradburd et al., 2016). SpaceMix creates a “geogenetic map” based on individuals' geography and genetics. Using Markov chain Monte Carlo (MCMC) simulations, individuals are placed on the map according to their geographic location. Next, the program moves individuals to another geogenetic position, based on the proportion of admixture they draw from other populations or individuals, and plots those locations on a map. For example, two populations that are genetically similar due to gene flow but geographically distant are positioned closer to one another in “geogenetic space” than they otherwise would be in geographic space alone. We used the most comprehensive model in which populations can choose their locations (move on a geogenetic map) and draw admixture. The convergence of the runs was checked using functions provided in the SpaceMix package. For each tested model, we ran 10 fast runs with 100,000 iterations and one long run with 50 million iterations, sampling the chain every 10,000 iterations and saving the model output every 100,000 iterations.

We estimated contemporary gene flow among five genetic clusters identified by ADMIXTURE and PCA (which conformed to sampled geographic regions) using a Bayesian MCMC analysis implemented in BayesAss3‐SNPs (Wilson and Rannala, 2003; Mussmann et al., 2019). We used 20 million iterations, discarding 1 million as burn‐in, and sampled every 1000 iterations for posterior densities of migration estimates. Sufficient mixing of chains and convergence of MCMC were determined using TRACER version 1.7.1 (Rambaut et al., 2018).

To further explore the genetic connectivity among populations, we estimated Patterson's *D* and f4‐ratio (ABBA‐BABA) using Dsuite (Malinsky et al., 2021), a program that estimates *D* and f4‐ratio statistics from biallelic SNPs across all trios of taxa present in the data set and an outgroup. This four‐taxon statistical test for admixture is also known as the ABBA BABA test (Durand et al., 2011; Malinsky et al., 2021). The ABBA pattern refers to P2 and P3 sharing the derived allele; whereas in the BABA pattern, the derived allele will be shared by P1 and P3. Under a scenario when introgression occurs between P3 and either P1 or P2, the frequency of ABBA or BABA patterns will be different, leading to a *D* significantly different from zero (Malinsky et al., 2021). *Agave aurea* samples were used as an outgroup for this analysis.

### Genetic diversity

Genetic diversity levels within *A. sobria* were estimated using multilocus heterozygosity with the R package inbreedR (Stoffel et al., 2016) and the inbreeding index Fhat3 using PLINK version 2.0 (Chang et al., 2015). We estimated the number of private alleles for each subspecies/geographic region using the R package poppr. We calculated unbiased average nucleotide diversity (π) within a window size of 100 bp in pixy version 1.2.7 (Korunes and Samuk, 2021). The input AllSites VCF file containing invariant and variant loci was generated using bcftools version 1.12 (Li, 2011). We also estimated the relatedness coefficient between each pair of individuals using the “relatedness2” function in VCFtools and the methodology described by Manichaikul et al. (2010).

### Isolation by distance and environment

IBD and isolation‐by‐environment (IBE) analyses were performed using Mantel and partial Mantel tests to evaluate the contribution of geography and environment in shaping genetic structure. We used a pairwise *F*_ST_/(1 − *F*_ST_) matrix for the genetic distance among sampling localities (Rousset, 1997). The geographic matrix was estimated using a Geographic Distance Matrix generator (https://github.com/persts/GeographicDistanceTools). For the environmental distance matrix, we obtained Euclidean distances of 19 bioclimate variables downloaded from WorldClim version 2 (Hijmans et al., 2005) for each locality using QGIS version 3.28 (QGIS.org, 2024). Simple and partial Mantel correlations were performed in the R package vegan with 9999 permutation tests (Oksanen et al., 2017).

### Genome scan for outlier loci

To assess the selection footprint, we used the Bayesian method implemented in BayeScan version 2.1 (Foll and Gaggiotti, 2008). BayeScan tests whether subpopulation‐specific allele frequencies, measured as *F*_ST_, are significantly different from the allele frequency within the shared gene pool and gives a posterior probability (alpha) to a model in which selection explains a difference in allele frequencies better than a null model. A positive alpha suggests directional selection, while a negative alpha suggests purifying or balancing selection. Given that BayeScan may suffer from elevated false‐positive rates under IBD and range expansion (Lotterhos and Whitlock, 2014) and that balancing or purifying selection is especially prone to such issues (Lotterhos and Whitlock, 2014), we focused on directional selection. BayeScan was run using prior odds of 100 (Lotterhos and Whitlock, 2014). A false discovery rate (FDR) of 0.05 was used, with the caveat that although this reduces the number of false positives, true selection signals may be missed (Foll and Gaggiotti, 2008). We also used a genome scan for selection as performed in the R package PCAdapt (Duforet‐Frebourg et al., 2014; Luu et al., 2016). The detection of outliers, the SNPs associated with selection, was established based on the vector of *z* scores when regressing SNPs with the principal components (using *K* = 4, as suggested by previous analyses of our data set). To determine the threshold of *p* values, we used Bonferroni correction.

Environmental and geographic variables showing significant associations with genetic distance in partial Mantel analyses (i.e., precipitation of wettest month, temperature annual range, mean diurnal range, and latitude) were further investigated by testing for signatures of local adaptation using redundancy analyses (RDA) and latent factor mixed models (LFMMs). LFMMs (Frichot et al., 2013) identify loci‐environment associations using a Bayesian mixed model with environmental variables included as fixed effects. Latent factors are derived from a PCA and used as random effects to control for population structure. We built the model using the lfmm_ridge function of the R package LFMM (Caye et al., 2019). The number of latent factors (*K* value) and regularization parameters (lambda) were set to 4 and 1e‐5 to minimize predictor error estimated by a cross‐validation method, as advised in the LFMM package manual. We calibrated *p* values using the genomic control method, and the false discovery rate (*q* value) was calculated following the Benjamini‐Hochberg procedure in the R package qvalue (Storey, 2003). Associations between an SNP and environmental factors with *q* < 0.01 were considered statistically supported.

We used RDA to identify the proportion of genetic variation explained by environmental predictors within a multivariate environment and detect the SNPs with significant association with environmental predictors. The RDA analysis was performed using the R package vegan version 2.5‐6 (Oksanen et al., 2017). Within each RDA, multicollinearity between environmental predictors was assessed using the R package psych version 1.9.12 (https://cran.r-project.org/web/packages/psych/index.html, cut‐off threshold at *r* = 0.7). The significance (at α = 0.05) of each full RDA model was assessed via ANOVA with 999 permutations, and variance inflation factors were evaluated for further evidence of multicollinearity between environmental predictors. RDA was also used to identify the SNP loadings in the ordination space to assess whether SNPs are associated with environmental predictors (i.e., SNPs under selection). Outlier SNPs were determined based on the distribution of SNP loadings on each significant RDA axis. SNPs that exhibit more than ±3 standard deviation (SD) from the mean loading were marked as putative outliers. This threshold was reported to minimize type I and II errors (Forester et al., 2018).

Loci identified as outliers by one of the tests were considered to be putatively under selection. To determine their identities, the sequences of transcriptome contigs containing putative outliers were compared to a custom database using the BLASTx search tool with a minimum *E* value of 1 × *e* ^−10^ (Altschul et al., 1990). The custom database included all the plant protein sequences available at Uniprot (https://www.uniprot.org/taxonomy?query=viridiplantae) and was constructed using the makeblastdb application (Camacho et al., 2009). We retrieved the gene ontology (GO) information from the UniProt database (https://www.uniprot.org/id-mapping) using UniProt IDs from the blast‐hit result. This information includes the biological processes, the molecular functions, and the cellular compartments associated with each gene product identified. After that, we used GOseq (Young et al., 2010) to conduct an enrichment analysis of GO terms, a method that accounts for the gene length bias in the detection of overrepresentation. The GO annotations for the whole transcriptome were used as background in the enrichment analysis.

Finally, to find out if the outlier SNPs would reveal a different genetic structure, we combined the identified outlier SNPs and used them to reconstruct the NJ tree among *A. sobria* individuals using the R package poppr. To determine if the observed pattern was explained by adaptive divergence and not by a different number of employed SNPs, we also extracted the three sets of random SNPs from the complete vcf file and repeated NJ tree reconstruction. The number of random SNPs was the same as that of identified outliers.

## RESULTS

We recovered 547,875 raw SNP genotypes. After filtering, we kept 73 individuals (64 for *A. sobria* complex and nine for *A. cerulata* ssp. *subcerulata*) genotyped with 8453 high‐quality SNPs. Five individuals from the *A. sobria* samples were removed due to the high proportion of missing data (>50%). All the multilocus genotypes were unique, suggesting that the data set had good discriminatory power and that no clones were sampled. The mean depth per individual was 54.1 (SD 12.3), whereas the average missingness per individual was 1.03%. The phylogenetic data set comprising 82 individuals of *A. sobria*, *A. cerulata* ssp. *subcerulata*, and *A. aurea* had 8507 SNPs.

### Phylogenetic analysis

**Figure 2:**
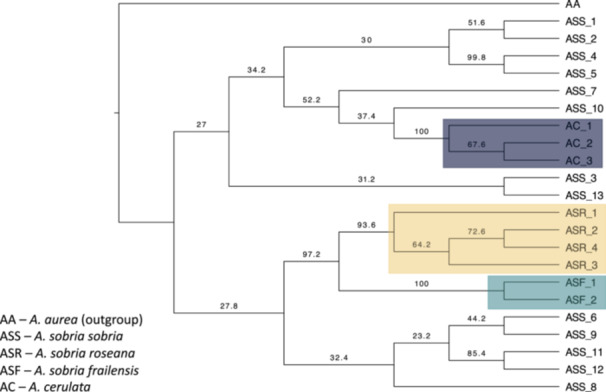
Phylogenetic tree constructed with the SVDquartets method in PAUP using 8453 SNPs. Numbers at nodes indicate bootstrap values (%). Colored blocks correspond to samples of *Agave sobria* ssp. *roseana* (orange), *A. cerulata* ssp. *subcerulata* (dark blue), and *A. sobria* ssp. *frailensis* (dark cyan). The tree was rooted using samples of *A. aurea*. Tips correspond to the sampling sites and are coded according to Appendix S1.

To elucidate taxonomic relationships within *A. sobria* subspecies, we constructed a phylogenetic tree based on 64 individuals of *A. sobria*, nine of *A. cerulata* ssp. *subcerulata*, and nine of *A. aurea*. Samples of *A. aurea* were used as an outgroup (Figure 2).

We found little evidence in support of the defined subspecies within *A. sobria* (Gentry, 1982; Webb and Starr, 2015). For example, *A. sobria* ssp*. sobria* did not form a monophyletic group; instead, samples of this subspecies were dispersed along the tree, reflecting geographic closeness. *Agave sobria* ssp. *frailensis* and *A. sobria* ssp. *roseana* each formed a well‐supported group. Nonetheless, they were part of one of the clusters, which also included southern *A. sobria* ssp. *sobria*. Moreover, the individuals of *A. cerulata* ssp. *subcerulata* included in the analysis did not seem to represent a differentiated species but were clustered with northern *A. sobria* ssp. *sobria*. In general, these results suggest that the tree topology is more consistent with ecological factors and the geographic distribution of the samples than with the morphologically recognized species and subspecies (Figure 2).

### Population genetic structure

**Figure 3:**
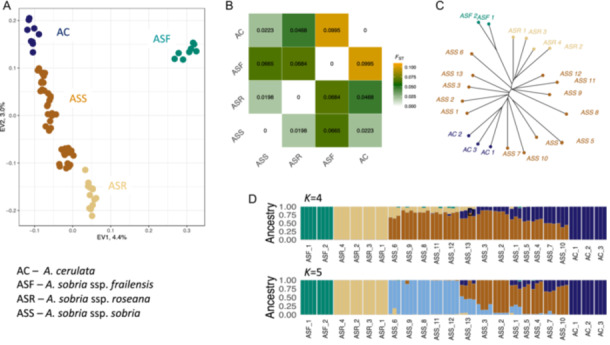
Population genetic structure of *Agave sobria* from the Baja California Peninsula (BCP), Mexico, based on 8453 genome‐wide SNPs. (A) Principal component analysis of the individuals of *A. sobria* and *A. cerulata* ssp. *subcerulata*. (B) Pairwise *F*_ST_ differences among *A. sobria* and *A. cerulata* ssp. *subcerulata* from the BCP. Colors represent *F*_ST_ values from the lowest (0) in white to the highest (0.1) in orange. (C) Neighbor‐joining (NJ) network reconstructed for *A. sobria* and *A. cerulata* ssp. *subcerulata* at the sampling site level. NJ tree tips are colored according to the three subspecies of *A. aurea* and *A. cerulata* ssp. *subcerulata*: green = *A. sobria* ssp. *frailensis*, light brown = *A. sobria* ssp. *roseana*, dark brown = *A. sobria* ssp. *sobria*, and dark blue = *A. cerulata* ssp. *subcerulata*. (D) Bar plots of the individual assignment probabilities (vertical axis) for the number of genetic clusters from *K* = 4 to *K* = 5 inferred using the program ADMIXTURE. Samples were clustered according to sampling sites and arranged from the southernmost sampling site (left) to the northernmost sites (right).

Population genetic structure analysis revealed some interesting patterns (Figure 3A). Principal component analysis (PCA) clustered *A. sobria* and *A. cerulata* ssp. *subcerulata* individuals into five genetic groups that reflected the geography of the area: (1) samples of *A. sobria* ssp. *frailensis*, the southernmost and the most divergent group; (2) samples of *A. sobria* ssp. *roseana*; (3) southern samples of *A. sobria* ssp. *sobria* (populations ASS 6, 8, 9, 11, 12); (4) samples of *A. sobria* ssp. *sobria* from northern Sierra La Giganta (populations ASS 1, 2, 3, 4, 5, 7, 10, 13); and (5) samples of *A. cerulata* ssp. *subcerulata*.

The genetic differentiation revealed by PCA within and among morphologically recognized subspecies of *A. sobria* and *A. cerulata* ssp. *subcerulata* and geographic regions was surprisingly low, as the PCA1 explained only 4.4% of the variance and the average was *F*_ST_ = 0.04 (Figure 3A, B).

The lowest differentiation at the taxa level was found between *A. sobria* ssp. *sobria* and *A. sobria* ssp. *roseana* (*F*_ST_ = 0.019), whereas the highest differentiation was found between *A. sobria* ssp. *frailensis* and *A. cerulata* ssp. *subcerulata* (*F*_ST_ = 0.099), the taxa with the largest geographic distance. Surprisingly, *A. cerulata* presented very low differentiation from the *A. sobria* subspecies, particularly from the geographically closed *A. sobria* ssp*. sobria* (*F*_ST_ = 0.022).

Similar results of relatively low differentiation were obtained when *F*_ST_ was estimated at the population level (Appendix S3) between pairs of localities, with the average *F*_ST_ = 0.05. The highest differentiation (*F*_ST_ = 0.123) was found between the southernmost site, *A. sobria* ssp. *frailensis* (ASF_2), and the *A. sobria* ssp. *sobria* site (ASS_10) from northern Sierra La Giganta, separated by ~390 km. The lowest differentiation (*F*_ST_ = 0.004) was found between two geographically close sites (ASR_3 and ASR_1) of *A. sobria* ssp. *roseana*, growing 11 km apart. Accordingly, the five genetic groups identified with PCA presented relatively low but significant divergence among them; for example, samples of southern and northern *A. sobria* ssp. *sobria* had *F*_ST_ = 0.012.

These findings of low overall differentiation were also reflected in the individual‐level and sampling‐site‐level NJ trees (Figure 3C; Appendix S4). However, as with PCA, it was possible to identify the five genetic clusters mentioned above. ADMIXTURE analysis confirmed low differentiation within and among *A. sobria* and *A. cerulata* samples. The cross‐validation error indicated that the most probable number of clusters within the analyzed samples was one (Appendix S5). When we plotted individuals' ancestry values for *K* = 2–5, we found the same grouping of the samples as inferred with PCA, with the majority of the individuals presenting high affinity to the corresponding cluster (Figure 3D, E; Appendix S6).

### Spatial genetic structure and migration

**Figure 4:**
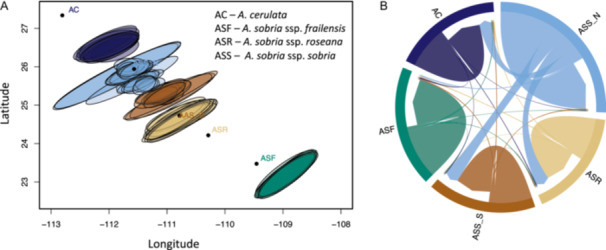
(A) Geogenetic map of the *Agave sobria* and *A. cerulata* ssp. *subcerulata* populations. The figure was inferred under the model with migration and admixture using SpaceMix software. Colored dots correspond to the averaged “true” geographic location of each population/subspecies. Ellipses show the 95% confidence surfaces of the estimated geogenetic location for each individual. Ellipses were colored according to the populations inferred with PCA and ADMIXTURE: dark cyan = *A. sobria* ssp. *frailensis*, light brown = *A. sobria* ssp. *roseana*, dark brown = southern *A. sobria* ssp. *sobria*, light blue = northern *A. sobria* ssp. *sobria*, and dark blue = *A. cerulata* ssp. *subcerulata*. (B) Circular plot of direction and intensity of recent migration flow among populations of *A. sobria* and *A. cerulata* ssp. *subcerulata*. Population codes: ASR = *A. sobria* ssp. *roseana*, ASF = *A. sobria* ssp. *frailensis*, AC = *A. cerulata* ssp. *subcerulata*, ASS‐N = *A. sobria* ssp. *sobria* north, and ASS‐S = *A. sobria* ssp. *sobria* south.

The geogenetic space estimated using SpaceMix for the *A. sobria* and *A. cerulata* (Figure 4) reflected a latitudinal distribution of the samples, similar to what was found with the PCA and the Mantel test, suggesting a strong effect of geography and neutral forces on these agave taxa. We also found that subspecies (i.e., except *A. sobria* ssp. *sobria*) presented low intragroup variation, as we observed almost a complete overlap of the samples (ellipses), suggesting relatively high relatedness of the individuals within each subspecies/region.

Moreover, each group occupied a different position in this geogenetic space compared to its actual geographic location. For example, the southernmost *A. sobria* ssp. *frailensis* was placed farther from the rest of the samples, suggesting that these samples are genetically more dissimilar than their geographic location suggests. By contrast, specimens of *A. sobria* ssp. *roseana*, *A. sobria* ssp. *sobria*, and *A. cerulata* ssp. *subcerulata* were positioned closer to one another in geogenetic space, suggesting that they are genetically closer than their geographic location suggests and possibly connected by a gene flow (Figure 4A).

The gene flow suggested by SpaceMix analysis was confirmed by the results of BA3‐SNPs (Figure 4B). We inferred unidirectional gene flow from the northern population *A. sobria* ssp. *sobria* to *A. cerulata*, southern *A. sobria* ssp. *sobria*, and *A. sobria* ssp. *roseana*. We did not find evidence for recent gene flow to or from *A. sobria* ssp. *frailensis*.

The DSuite ABBA‐BABA analyses confirmed the gene flow patterns inferred with BA3 and SpaceMix (Appendix S7; Figure 4). The highest values of the f4‐ratio and lowest *p* values were found between the southern and northern populations of *A. sobria* ssp. *sobria*, and between *A. sobria* ssp. *roseana* and both populations of *A. sobria* ssp. *sobria*; whereas insignificant values were found for the geographically distant populations (e.g., *A. sobria* ssp. *sobria* vs. *A. sobria* ssp. *frailensis*).

### Genomic diversity

Based on 8453 SNPs, the individual‐based multilocus heterozygosity (MLH) was relatively high (0.26, SD 0.02), the highest MLH being found in an individual from the ASS_12 population (MLH = 0.31), whereas the lowest was in an individual from the ASF_1 population (MLH = 0.20). At the subspecies level, the highest MLH was found in *A. sobria* ssp. *sobria* (0.28, SD 0.009), and the lowest values were obtained for *A. sobria* ssp. *frailensis* (0.24, SD 0.017; Table 1).

**Table 1:** Diversity estimates for the samples of *Agave sobria* complex and *Agave cerulata* ssp. *subcerulata* (*n* = number of samples; *H*_o_ = observed heterozygosity; *H*_e_ = expected heterozygosity; *F*_IS_ = Wright's inbreeding index; Fhat3 = inbreeding index; MLH = multilocus heterozygosity; *P*_*i*_ = unbiased nucleotide diversity). Standard deviation is in parentheses.

Subspecies	*n*	Private alleles	*H*_o_	*H*_e_	*F*_IS_	Fhat3	MLH	*P*_*i*_
*A. sobria ssp. frailensis*	8	8	0.24 (0.27)	0.19 (0.19)	−0.03 (0.07)	−0.03 (0.04)	0.24 (0.007)	0.064 (0.12)
*A. sobria ssp. roseana*	13	4	0.27 (0.23)	0.22 (0.16)	−0.15 (0.07)	−0.09 (0.03)	0.27 (0.02)	0.074 (0.12)
*A. sobria ssp. sobria*	43	198	0.28 (0.20)	0.24 (0.14)	−0.19 (0.04)	−0.12 (0.02)	0.28 (0.009)	0.077 (0.11)
*A. cerulata ssp. subcerulata*	9	2	0.27 (0.24)	0.22 (0.17)	−0.16 (0.03)	−0.11 (0.02)	0.27 (0.006)	0.077 (0.1)

We found an overall significant (Bartlett's test, *p* < 0.001) excess of heterozygosity (*H*_O_: 0.27, SD 0.19; *H*_E_: 0. 24, SD 0.13). The same pattern of heterozygote excess persisted within each subspecies (Table 1). The heterozygote excess was also expressed in an average negative *F*_IS_ index (−0.16, SD 0.07), which was lowest in *A. sobria* ssp. *sobria* (*F*_IS_ = −0.16, SD 0.04) and the highest in *A. sobria* ssp. *frailensis* (*F*_IS_ = −0.03, SD 0.04). Like *F*_IS_, the Fhat3 inbreeding index was negative in *A. sobria* (Table 1).

The nucleotide diversity differed among subspecies, with the highest values found in *A. sobria* ssp. *sobria* and *A. cerulata* ssp. *subcerulata* (0.077, SD 0.1 for both subspecies) and the lowest values (0.064, SD 0.12) in *A. sobria* ssp. *frailensis*. Each analyzed taxon had private alleles. The lowest number (2) was found in *A. cerulata*, and the highest for *A. sobria* ssp. *sobria*, with 198 private alleles (Table 1).

The average relatedness among all the samples was high (0.132, SD 0.03), corresponding to the second‐degree relationships among specimens. As expected, relatedness was higher within each subspecies compared to between subspecies (Appendix S8). Within each taxon, the lowest average relatedness was found among individuals from *A. sobria* ssp. *sobria* (0.15, SD 0.01) and *A. sobria* ssp. *roseana* (0.15, SD 0.02). The highest relatedness (0.17, SD 0.03) was inferred among samples of *A. sobria* ssp. *frailensis*; the same levels of relatedness were also estimated among samples of *A. cerulata* ssp. *subcerulata* (Appendix S8).

### Isolation by distance and by environment

Mantel tests revealed positive correlations between linearized genetic and geographic distance for *A. sobria* and *A. cerulata* ssp. *subcerulata* samples (Mantel's *r* = 0.59, *p* < 0.0001; Appendices S9, S10).

Furthermore, partial Mantel tests revealed significant associations between genetic distance and several environmental variables, even after controlling for geographic distance (Appendix S10). Specifically, the mean diurnal range (BIO2) and temperature annual range (BIO7) were significantly correlated with genetic distance in *A. sobria* (partial Mantel test, *r* = 0.56, *p* < 0.001, and *r* = 0.45, *p* < 0.001, respectively).

### Genome scan for outlier loci

**Figure 5:**
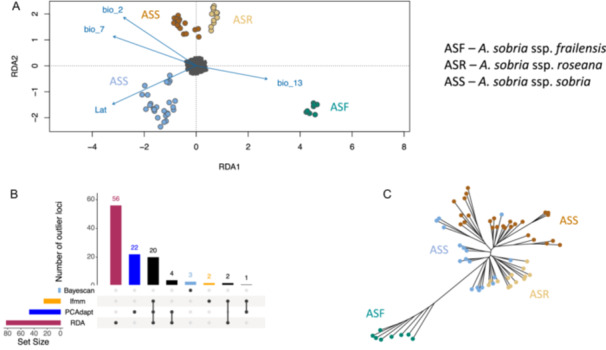
(A) Redundancy analysis (RDA) showing the relative contributions of environmental and geographic variables to the genetic structure of outlier and neutral genotypes. SNP genotypes are in gray; individuals are represented by different colors according to their location. Plot shows the most relevant variables obtained with a partial Mantel test. (B) Upset plot of the number of putatively adaptive loci detected for *Agave sobria* by BayeScan, PCAdapt, LFMM, and RDA. The figure indicates the exclusive loci detected by each method and the common ones shared between two or three methods. (C) Phylogenetic networks constructed for *A. sobria* using outlier loci exclusively.

To investigate the basis of the associations between genomic divergence and ecological variables, we used four complementary approaches. PCAdapt and BayeScan identified 47 and three loci as “outlier” markers, respectively (Figure 5). The other two methods (RDA and LFMMs) explicitly considered ecological and spatial data and searched for genotype‐environmental associations. After correcting the corresponding *p* values (0.01), the LFMMs detected 25 SNPs exhibiting association with mean diurnal range, temperature annual range, precipitation of wettest month, and latitude in *A. sobria* (Figure 5B). We used a multivariate linear regression approach implemented in RDA to test for genotype‐environment associations (Figure 5A). The full RDA model was significant (*p* = 0.001) and explained 5.2% of the observed variance. RDA identified all four axes as significant (*p* = 0.001 in all cases). The outlier analysis on the significant RDA axes detected 82 significant SNPs (Figure 5B).

In total, we identified 110 outlier SNPs (Figure 5B). Although some outlier SNPs were detected by only one method, we found 20 SNPs that were identified by the three analyses (RDA, PCAdapt, and LFMMs) and seven additional markers identified by a combination of two methods (Figure 5B).

We used the UniProt (https://www.uniprot.org) plant protein database to functionally annotate the transcripts containing the outlier markers using Blastx (Appendix S11). The species with the major number of blast hits was *Asparagus officinalis* (Appendix S11). An enrichment analysis of GO terms carried out against a background list of GO‐annotated sequences of the *A. sobria* transcriptome revealed 39 significantly enriched GO terms (19 biological processes, 16 molecular functions, and four cellular compartments). Among them, we found biological processes related to cold acclimation, detection of visible light, and response to ionizing radiation that may be important to local adaptation (Appendix S12).

Finally, we asked whether the total number of outlier loci identified by all the methods (*n* = 110) would resolve contrasting phylogenies to the neutral loci (defined as those loci that were not identified by either program). The recovered NJ tree revealed strong support for the presence of two genetic clades (Figure 5C), one corresponding to *A. sobria* ssp. *frailensis* and another to *A. sobria* ssp. *sobria* and *A. sobria* ssp. *reseana*. Moreover, the outlier loci exhibit stronger differentiation between *A. sobria* ssp. *frailensis* and the rest of the samples, compared to 110 randomly sampled neutral loci (Appendix S13) and the complete data set (Appendix S4).

## DISCUSSION

### Subspecies status within *Agave sobria*

One of the main objectives of our study was to resolve genetic relationships within *A. sobria*. This is one of the most morphologically variable agave species in Baja California, at least based on its current conception and morphological characteristics (see Webb and Starr, 2015). There are three recognized subspecies of *A. sobria*. However, the designations have been based on morphological data alone, leading to unclear subspecies distribution limits and possible mistakes during identification due to the similarity of the morphology (Gentry, 1978; Webb and Starr, 2015).

Our genome‐wide data set, including over 8000 SNPs genotyped in 64 individuals from 19 locations covering the complete distribution range of *A. sobria* (Appendix S1; Figure 1), contradicts the alleged separation between subspecies (Webb and Starr, 2015). Our data suggest that the morphological characteristics used as a basis for subspecies delineation are better explained, at least in part, by phenotypic plasticity and geographic distance than by reproductive isolation. The phylogenetic analysis unraveled several patterns and showed low support for some of the recognized taxonomic relationships within *A. sobria*.

Our findings are not unexpected, given that prior research on Baja California agaves found that genetic distances correlate poorly with taxonomic divisions (Navarro‐Quezada et al., 2003; Klimova et al., 2024). Furthermore, the relationships within *Agave* have been difficult to resolve at the species level, with the recovered species polytomies probably related to rapid radiation and gene flow among subspecies (ongoing hybridization; Jiménez‐Barron et al., 2020; Heyduk et al., 2016, 2023).

The two main clades detected by the SVDquartets analysis reflected eco‐geographic divergence, placing southern samples (*A. sobria* ssp. *frailensis*, *A. sobria* ssp. *roseana*, and southern *A. sobria* ssp. *sobria*) into a group sister to all the northern samples of *A. sobria* ssp. *sobria* and *A. cerulata* ssp. *subcerulata*. Moreover, branch support was relatively low, even for the main groups. *Agave sobria* ssp. *sobria* appears as polyphyletic because its populations were grouped in different clades in the tree. Within the southern clade, *A. sobria* ssp. *frailensis* and *A. sobria* ssp. *roseana* each formed well‐supported groups. However, this clustering was related to the ecology and geography of the peninsula rather than to the species/subspecies divergence (see discussion on population structure below).

We also included nine individuals from three populations of *A. cerulata* ssp. *subcerulata* to be used as an outgroup for *A. sobria* samples. *Agave cerulata* ssp. *subcerulata* occurs mainly in Baja California Sur state, occupying roughly the central part of the peninsula, and its distribution does not overlap with the *A. sobria* range (Navarro‐Quezada et al., 2003; Webb and Starr, 2015). Although *A. cerulata* ssp. *subcerulata* is considered a different species and is morphologically differentiated from *A. sobria*, it was suggested that it may have a genetic connection to some populations of *A. sobria* (Gentry, 1978; Webb and Starr, 2015). We confirmed these suggestions, as phylogenetic analysis placed *A. cerulata* ssp. *subcerulata* samples within the northern clade of *A. sobria* samples.

Further studies should use genomic and morphological information to revise and clarify species and subspecies designation in the whole Deserticolae Section *sensu* Gentry (1978, 1982), including all the different subspecies and taxa of *A. cerulata*, *A. sobria*, *A. deserti*, *A. subsimplex*, and *A. pringlei* (Navarro‐Quezada et al., 2003; Webb and Starr, 2015).

### Levels of genetic variation

Genomic variation in *A. sobria* (MLH = 0.26, SD 0.02) was higher than the values estimated using similar genomic methods (i.e., genotyping‐by‐sequencing) in other wild agave species: *A. aurea* (MLH = 0.22; Klimova et al., 2024), wild *A. angustifolia* (MLH = 0.22; Klimova et al., 2022), *A. potatorum* (MLH = 0.21; Ruiz‐Mondragón et al., 2023), or *A. marmorata* (MLH = 0.19; Ruiz‐Mondragón et al., 2024).

High genetic diversity in *A. sobria* is not surprising, and it is in concordance with the ecology and life history of the genus *Agave*: long‐lived perennial plants, mostly outcrossing ones whose pollinators (bats and birds) are highly mobile and thus capable of long‐distance pollen dispersal (Loveless and Hamrick, 1984; Eguiarte et al., 2013, 2021). The high levels of genetic diversity within *A. sobria* seems to be related not only to its ecology, but also to a considerable gene flow from populations at the northern extreme of the species distribution range, according to SpaceMix, Dsuite, and BA3‐SNPs analyses.

However, the distribution of genetic diversity among geographic regions was not even. We found that *A. sobria* ssp. *frailensis* (the southernmost taxa) had significantly lower diversity (heterozygosity and nucleotide diversity) than other populations. These findings may be explained by the geographic isolation of southernmost samples and the lack of gene flow, which may have promoted genetic drift in this area or by the local adaptation in this subspecies (see below).

Interestingly, along with high genetic diversity (heterozygosity), we also observed heterozygote excess, negative inbreeding indices (*F*_IS_ and Fhat), and relatively high relatedness among individuals. Heterozygote excess in natural plant populations is common and has several possible causes (Eguiarte et al., 1992; Balloux, 2004; Stoeckel et al., 2006). For instance, if only a few breeders contribute to the next generation, allelic frequencies can differ between male and female parents by chance, leading to heterozygote excess in their progeny (Rasmussen, 1979; Pudovkin et al., 1996). However, we do not think that in *A. sobria* negative *F*_IS_ could result from a small number of breeders. *Agave sobria* is not endangered; it has relatively healthy populations and is scattered but common on sierras and hills in the peninsula's southern half. During our field trips, we also observed a high number of flowering mature plants and many young individuals.

Another possible explanation for negative *F*_IS_ and heterozygote excess involves asexual reproduction that maintains heterozygosity and increases it by accumulating somatic mutation over generations (Balloux et al., 2003, 2004). An increase in heterozygosity has been observed in many natural plant species, in particular in populations that reproduce clonally (Balloux, 2004; Stoeckel et al., 2006; Da Cunha et al., 2022), and is common in clonally propagated crops (e.g., potatoes, grapes, cassava; McKey et al., 2010; Ghislain and Douches, 2020; Qi et al., 2022).

The genomic variation in *A. sobria* (i.e., heterozygotes excess, negative inbreeding indices, and relatively high relatedness) is similar to the patterns detected in cultivated, clonally propagated *A. tequilana* and *A. angustifolia* (Cabrera‐Toledo et al., 2022; Ruiz‐Mondragon et al., 2022; Klimova et al., 2023). By contrast, sexually reproducing wild populations of Agavoideae (i.e., agaves and yuccas) usually display moderate levels of inbreeding, heterozygote deficiency, and low relatedness, as was found in *A. aurea*, *A. marmorata*, *A. potatorum*, *A. angustifolia*, *Yucca valida* Brandegee, and *Y. capensis* L.W.Lenz (Arteaga et al., 2020; Klimova et al., 2021; Ruiz‐Mondragón et al., 2023; Klimova et al., 2024; Ruiz‐Mondragón et al., 2024). These findings may suggest that clonal propagation is common in *A. sobria*.

Clonality in *A. sobria* could be an adaptation to harsh environmental conditions in which sexual recruitment is restricted to “good” (usually more humid) years (Jordan and Nobel, 1979; Vallejo‐Marín et al., 2010; Barrett, 2015). In support of this hypothesis, we found that clonal reproduction seems to be more common in northern populations (more negative inbreeding and pronounced heterozygote excess), where the climate is drier and colder than in southern populations (Webb and Starr, 2015; A. Klimova, personal observation).

### Population structure and migration

Previous descriptions based on morphology indicated that the *A. sobria* complex is a morphologically diverse group, potentially containing three subspecies (Gentry, 1978; Webb and Starr, 2015). However, our genomic data set provides a more nuanced understanding. It supports the reclassification of *A. sobria* as a single species and further reveals that it comprises four distinct but genetically close intraspecific lineages. We found that various factors, including high habitat heterogeneity, latitudinal clines, and ecological factors related to temperature and precipitation, seem to influence the differentiation of these lineages.

*Agave sobria* populations showed relatively weak genetic structure but clear IBD pattern, with PCA almost perfectly reflecting the geographic distribution of the samples. Genetic differentiation was low within *A. sobria* (average paired *F*_ST_ = 0.04). The magnitude of this differentiation is very similar to that reported in other agaves (Eguiarte et al., 2013). Wild agaves, in general, present low intraspecific differentiation, as was found in wild *A. angustifolia* (*F*_ST_ = 0.076; Klimova et al., 2022), *A. desertia* (*F*_ST_ = 0.135; Navarro‐Quezada et al., 2003), *A. cerulata* (*F*_ST_ = 0.098; Navarro‐Quezada et al., 2003), *A. marmorata* (*F*_ST_ = 0.087; Ruiz‐Mondragón et al., 2024), *A. potatorum* (*F*_ST_ = 0.079; Ruiz‐Mondragón et al., 2023), and *A. aurea* (*F*_ST_ = 0.03–0.14; Klimova et al., 2024). This low differentiation may be explained by the high genetic connectivity promoted by the primary agave pollinators, nectarivorous bats in the genus *Leptonycteris* (Eguiarte et al., 2021; Alducin‐Martínez et al., 2022). Although the pollination biology of *A. sobria* is still unknown and needs further investigation, several lines of evidence suggest that gene flow is widespread in *A. sobria*. Within the distribution range of *A. sobria*, a nectar‐feeding bat (*Leptonycteris yerbabuenae*) can be found; this bat species has a panmictic population structure, indicating a high level of gene flow among colonies (Arteaga et al., 2018). It is, therefore, highly possible that bats feed on *A. sobria* flowers and move pollen from population to population.

We used three complementary approaches to estimate the level and directions of gene flow and admixture in the *A. sobria* population. The BA3 analysis, which reflects recent migration, indicated strong unidirectional gene flow from the northern population of *A. sobria* ssp. *sobria* to the north (to *A. cerulata* ssp. *subcerulata*) and to the south (to southern *A. sobria* ssp. *sobria* and *A. sobria* ssp. *roseana*). The geogenetic map reconstructed with SpaceMix showed that the geogenetic distances among samples were distorted (compared with the geographic distances) due to gene flow between *A. cerulata* ssp. *subcerulata*, *A. sobria* ssp. *sobria*, and *A. sobria* ssp. *roseana*. Patterson's *D*, f‐4 ratio test, and ADMIXTURE (*K* = 4 and *K* = 5) also suggested extensive gene flow and admixture between samples of *A. sobria* ssp. *sobria*, *A. sobria* ssp. *roseana*, and *A. cerulata* ssp. *subcerulata*.

These findings align with previous studies on *A. sobria* relatives (*A. deserti* and *A. cerulata*), where a pattern of IBD and gene flow among populations was found (Navarro‐Quezada et al., 2003). The strong IBD found for *A. sobria* seems to be the result of two opposite forces: on one side, the extensive gene flow, probably promoted by pollinators; and on the other side, the clonal reproduction that enhances the drift by reducing the opportunity for genetic recombination (Milgroom, 1996).

Although gene flow is a relevant factor in genetic homogenization in *A. sobria* and *A. cerulata* ssp. *subcerulata*, we also detected significant structuring within *A. sobria*. For example, the southernmost subspecies/population of *A. sobria* ssp. *frailensis* was strongly differentiated from the rest of the samples. This divergence may be explained by the geographic isolation of this subspecies, whose available habitat is surrounded by low‐elevation sandy desert areas. The resulting geographic isolation may enhance genetic drift and the loss of genetic diversity in this subspecies. However, IBE also seems to be a crucial factor in shaping structure and diversity in *A. sobria* ssp. *frailensis* (see above).

### Ecologically mediated divergence

The primary purpose of population genetic studies is to understand the underlying causes and biological nature of the variation in traits and genomic divergence among populations so that this information can be used to predict responses to future conditions and understand the basis of speciation (Okazaki et al., 2020; Hamilton, 2021). Genome‐environmental association studies in wild agave populations are still challenging due to many factors, such as a lack of baseline studies and complex genetic interactions (Eguiarte et al., 2021; Davis and Ortiz‐Cano, 2023; Yang et al., 2023). The influence of genes and environment on important traits remains elusive, even in cultivated and economically important agave species, let alone in wild and understudied taxa (Ruiz‐Mondragon et al., 2022; Klimova et al., 2023).

However, agaves are attractive as a case study because closely related species/populations may occupy contrasting environments and latitudinal or altitudinal gradients (Gentry, 1982). Agaves flourish in arid conditions, making them an important model for understanding species' responses and adaptation to ongoing climate change (Davis and Ortiz‐Cano, 2023; Yang et al., 2023). Moreover, many agave species are of social and economic importance, and several species are subject to ongoing domestication (Eguiarte et al., 2021; Jimenez‐Torres et al., 2021; Ruiz‐Mondragon et al., 2022; Klimova et al., 2023).

*Agave sobria* occupies a considerable latitudinal gradient (>400 km), with relevant temperature and precipitation differences between the northernmost and southernmost distribution extremes (Webb and Starr, 2015). Under such conditions, local adaptation should be relevant in shaping divergence patterns across the genome (Halbritter et al., 2015; Chung et al., 2023). Our results are consistent with this idea and imply that local adaptation may have contributed to the population structure of *A. sobria*, particularly in its southernmost subspecies, *A. sobria* ssp. *frailensis*. We detected strong and significant associations between several environmental variables and genetic distance in *A. sobria*. Genetic distance was correlated to the mean diurnal range, temperature annual range, isothermality, precipitation in the wettest month, and maximum temperature of the warmest month.

To further explore these findings, we used two approaches to detect outlier loci without incorporating environmental variables and two methods that explicitly search for loci showing significant associations with key ecological parameters. We tried to reduce the occurrence of false positives, controlling for the underlying population structure, which diminishes the occurrence of spurious genotypes by environmental associations (Salloum et al., 2022). Each approach identified a different number of outlier loci, but 7 markers were identified by different combinations of the two methods, and 20 markers were identified by three methods.

To capture as many outliers as possible, we pooled all the loci flagged by at least one approach and classified the remaining loci as putatively neutral. Constructing phylogenetic trees separately for these two classes of markers revealed a clear differentiation of *A. sobria* ssp. *frailensis* (Figure 5) from the rest of the samples, whereas the same number of randomly sampled neutral loci failed to resolve any structure within *A. sobria* (Appendix S13). These findings are reminiscent of similar studies that resolved different trees based on neutral and outlier loci (Matala et al., 2014; Funk et al., 2016; Klimova et al., 2017; Barbosa et al., 2021). Such a pattern could be considered a footprint of selection, as *A. sobria* ssp. *frailensis* and other subspecies presented similar genotypes at the neutral loci, while diversifying selection at outlier loci resulted in strong divergence between these groups.

Further insights were gained when functional annotation of the transcripts containing outliers was obtained. We found several genes whose protein products have interesting functions related to environmental adaptation. Among them are Zinc‐Finger type proteins implicated in plant tolerance to abiotic stress factors (e.g., salt, drought, flooding, cold temperatures, and oxidative stress; Agarwal and Khurana, 2018; Han et al., 2021, 2022). Additional outlier markers were associated with genes encoding proteins and transcription factors involved in stress response and drought tolerance. Among these genes we found the thaumatin‐like protein, ABC transporter domain‐containing protein, BHLH transcription factor, calcium‐transporting ATPase, DUF4228 domain‐containing protein, and DUF1645 domain‐containing protein (Brandazza et al., 2004; Kang et al., 2011; Cui et al., 2016; Guo et al., 2021; Lv et al., 2022; Park and Shin, 2022). We also found outliers in genes encoding the WEB family proteins, which are required for the chloroplast avoidance response under high‐intensity blue light, which may be useful as an adaptation to high solar radiation (Kodama et al., 2010; Kang et al., 2011).

Besides environmental divergence, pathogens may also play a role in ecological speciation (Gilbert, 2002; Giraud et al., 2010). It is possible that differences in the immunological challenges encountered within the contrasting environments where *A. sobria* ssp. *frailensis* and *A. sobria* are found might have shaped patterns of genetic divergence between these two groups. Accordingly, we found outliers on genes encoding the NDR1/HIN1‐like protein 6, which plays a vital function in pathogen‐induced plant responses to biotic stress (Bao et al., 2016), RPM1 interacting protein 13, that enhances resistance to pathogenic bacteria (Liu et al., 2020), RLK, which appears to play a main function in signaling during pathogen recognition and the subsequent activation of plant defense mechanisms (Afzal et al., 2008), and flavin‐containing monooxygenase involved in pathogen defense (Schlaich, 2007).

We also found statistical significance for genomic enrichment in several biological processes related to responses to abiotic stress (i.e., response to ionizing radiation, cold acclimation, and detection of visible light). Further studies should delve further into the differential gene expression of plants found, for example, using RNA‐seq and confirming the function of the transcripts carrying the identified outliers.

### Implications for conservation

The flora of the BCP, like most biodiversity on Earth, is under serious threat because of human activities, which include climate change, habitat fragmentation, overexploitation, invasive species, and free‐roaming domestic animals, among others (Tilman and Lehman, 2001; Dirzo et al., 2014; Wehncke et al., 2014; Corlett, 2016; Zhang et al., 2023). The flora and fauna of arid and semiarid areas are particularly vulnerable to ongoing climate change (Hantson et al., 2021; Zhang et al., 2023). In this scenario, understanding the distribution of genetic variation across spatial scales and its causal mechanism becomes crucial for biodiversity conservation.

The establishment of conservation and management units should be based on evidence that considers population differentiation, genetic diversity, landscape connectivity, and adaptive divergence (Mable, 2018; Hohenlohe et al., 2021). Using genomic SNPs, we identified five groups within two main genetic clusters: *A. cerulata* ssp. *subcerulata*, northern *A. sobria* ssp. *sobria*, southern *A. sobria* ssp. *sobria*, *A. sobria* ssp. *roseana*, and *A. sobria* ssp. *frailensis*. Some of these genetic groups, particularly northern ones, are well connected by gene flow, which may make them more resilient.

*Agave sobria* ssp. *frailensis*, on the other hand, is genetically different, with no genetic connectivity to other *A. sobria* populations, and exhibits adaptation to the local environment. Therefore, conservation efforts should be focused on maintaining its respective genetic diversity, especially considering that it occupies only a very small geographic area (i.e., several hills in the southern tip of the BCP; Figure 1).

Moreover, given that clonal propagation seems to be widespread in *A. sobria*, it will be important to understand the factors involved in gene flow among populations. Particular attention should be paid to studies of the biological interactions between agaves and their primary pollinators (bats, moths, birds, bees), whose conservation is expected to increase gene flow and, by increasing genetic variation, to facilitate local adaptation to cope with a rapidly changing climate (Gómez‐Ruiz and Lacher, 2019; Trejo‐Salazar et al., 2023).

## CONCLUSIONS

Speciation is a complex process that requires understanding interactions between local adaptation, phenotypic plasticity, evolutionary potential, effective population size, dispersal ability, and interspecific interactions. Our data provide compelling evidence that *A. sobria* is experiencing adaptive differentiation driven by a heterogeneous environment, restricted gene flow, and high genetic and morphological diversity. The climatic conditions influencing the southern *A. sobria* ssp. *frailensis* lineage are different from those governing the rest of the populations, which promotes ecological divergence. Meanwhile, the divergence of other subspecies and populations seems to be constrained by gene flow that, combined with clonal reproduction and the peculiar geography of the BCP, promotes IBD.

## AUTHOR CONTRIBUTIONS

Conceptualization: L.E.E. and A.K. Sample collection and data curation: A.K. and J.G.R. Formal analysis: A.K., O.E.J., and J.G.R. Funding acquisition: L.E.E. and A.O.R. Methodology: A.K. Supervision: L.E.E. and A.O.R. Writing—original draft preparation: A.K. Writing—review and editing: L.E.E., A.K., O.E.J., and J.G.R. All authors have read and agreed to the published version of the manuscript.

## Supporting information


**Appendix S1.** Collection sites for 73 individuals of three subspecies of *Agave sobria* and *Agave cerulata* ssp. *subcerulata* from the BCP.

**Appendix S2.** Summarized flow chart, including the analyses and programs used for neutral and adaptive approaches.

**Appendix S3.** Pairwise FST differences among all the sampling sites of three subspecies of *Agave sobria* and *A. cerulata* spp. *subcerulata* from the BCP.

**Appendix S4.** Individual‐based NJ phylogenetic network of *A. sobria* subspecies and *A. cerulata subcerulata*.

**Appendix S5.** A plot of ADMIXTURE cross‐validation error and respective standard deviation based on 20 repetitions for each *K* value, from *K* = 1 through *K* = 10, based on all samples of *A. sobria* and *A. cerulata* and 8453 SNPs.

**Appendix S6.** Population genetic structure of the *A. sobria* and *A. cerulata* ssp. *subcerulata* samples collected on the BCP, based on 8453 SNPs.

**Appendix S7.** Gene flow tests between *A. sobria* and *A. cerulata* ssp. *subcerulata* regions/populations.

**Appendix S8.** The relatedness estimates for within and between subspecies of samples belonging to *A. sobria* and *A. cerulata* ssp. *subcerulata* subspecies collected on the BCP.

**Appendix S9.** Mantel test results show the relationships between geographic distance and genetic distance as estimates with pairwise *F*_ST_ among sampling sites of *A. sobria* and *A. cerulata* ssp. *subcerulata* from the BCP.

**Appendix S10.** Mantel and partial Mantel tests summarizing relationships (*r* and associated *p* values) between genetic distance, geographic distance, and climate variables in *A. sobria*.

**Appendix S11.** Results of BLASTx analysis of transcripts containing putative outlier loci.

**Appendix S12.** List of significantly enriched gene ontology (GO) terms for transcripts of *A. sobria* containing outlier markers.

**Appendix S13.** NJ phylogenetic networks of individuals of *A. sobria* based on 110 random SNPs.

## Data Availability

The data have been deposited in the Sequence Read Archive of NCBI under the numbers SRR32693476 (https://www.ncbi.nlm.nih.gov/sra/?term=SRR32693476), SRR32693477 (https://www.ncbi.nlm.nih.gov/sra/?term=SRR32693477), and, SRR32693478 (https://www.ncbi.nlm.nih.gov/sra/?term=SRR32693478).
