# Supplementary material for: The interplay among space, environment, and gene flow drives genetic differentiation in endemic Baja California Agave sobria subspecies
Source: Am J Bot. 2025 Jul 2;112(7):e70062. doi: 10.1002/ajb2.70062 (PMC12281270; doi:10.1002/ajb2.70062)
Supplement: Supplementary file 1 — Appendix S1. Collection sites for 73 individuals of three subspecies of Agave sobria and Agave cerulata ssp. subcerulata from the BCP. [file AJB2-112-e70062-s008.pdf]

Appendix S1. Collection sites for 73 individuals of three subspecies of *Agave sobria* and *Agave cerulata* ssp. *subcerulata* from the Baja California Peninsula, Mexico., SG_N - Sierra La Giganta north, SG_S – Sierra La Giganta south, SFS - Sierra San Francisco, Cape_N - Cape region North, and Cape_S – Cape region south. N - number of individuals used for genomic analyses. Samples were collected in 2023.

Id	Subspecies	Region	Site name	Latitude	Longitude	N
1	*A.sobria* ssp. *sobria*	SG_N	ASS_1	25.971035	-111.48475	3
2	*A. sobria* ssp. *roseana*	Cape_N	ASR_1	24.328512	-110.31812	3
3	*A. sobria* ssp. *sobria*	SG_N	ASS_2	25.915204	-111.55073	4
4	*A. sobria* ssp. *sobria*	SG_N	ASS_3	25.657597	-111.25454	4
5	*A. sobria* ssp. *sobria*	SG_N	ASS_4	26.056265	-111.83049	3
6	*A. sobria* ssp. *sobria*	SG_N	ASS_5	26.004215	-111.87215	2
7	*A. sobria* ssp. *sobria*	SG_S	ASS_6	24.282699	-110.62867	3
8	*A. sobria* ssp. *roseana*	Cape_N	ASR_2	24.185281	-110.29988	3
9	*A. sobria* ssp. *frailensis*	Cape_S	ASF_1	23.446802	-109.43304	4
10	*A. sobria* ssp. *sobria*	SG_N	ASS_7	26.213196	-112.02183	3
11	*A. sobria* ssp. *sobria*	SG_S	ASS_8	24.821212	-110.81877	3
12	*A. sobria* ssp. *sobria*	SG_S	ASS_9	24.802387	-110.66674	4
13	*A. sobria* ssp. *frailensis*	Cape_S	ASF_2	23.500873	-109.47975	4
14	*A. sobria* ssp. *roseana*	Cape_N	ASR_3	24.248114	-110.24749	3
15	*A. sobria* ssp. *roseana*	Cape_N	ASR_4	24.126837	-110.28994	4
16	*A. sobria* ssp. *sobria*	SG_N	ASS_10	26.393733	-111.68015	3
17	*A.cerulata* ssp. *subcerulata*	SFS	AC_1	27.315613	-112.83384	3
18	*A.cerulata* ssp. *subcerulata*	SFS	AC_2	27.292671	-113.03433	3
19	*A.cerulata* ssp. *subcerulata*	SFS	AC_3	27.406511	-112.55183	3
20	*A. sobria* ssp. *sobria*	SG_S	ASS_11	24.833533	-110.90853	3
21	*A. sobria* ssp. *sobria*	SG_S	ASS_12	24.834326	-110.89891	4
22	*A. sobria* ssp. *sobria*	SG_N	ASS_13	25.564287	-111.19094	4
