# Supplementary material for: The interplay among space, environment, and gene flow drives genetic differentiation in endemic Baja California Agave sobria subspecies
Source: Am J Bot. 2025 Jul 2;112(7):e70062. doi: 10.1002/ajb2.70062 (PMC12281270; doi:10.1002/ajb2.70062)

Appendix S2. Summarized flow chart, including the analyses and programs used for neutral and adaptive approaches.

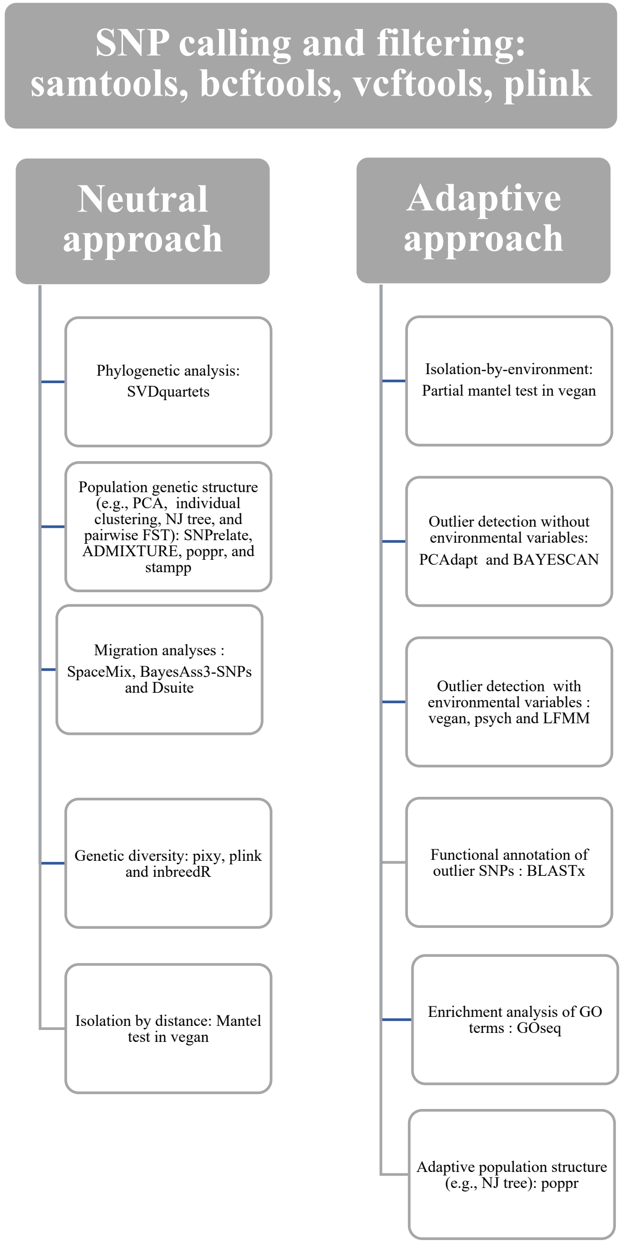

Supplement: Supplementary file 2 — Appendix S2. Summarized flow chart, including the analyses and programs used for neutral and adaptive approaches. [file AJB2-112-e70062-s009.pdf]
