# Supplementary material for: The interplay among space, environment, and gene flow drives genetic differentiation in endemic Baja California Agave sobria subspecies
Source: Am J Bot. 2025 Jul 2;112(7):e70062. doi: 10.1002/ajb2.70062 (PMC12281270; doi:10.1002/ajb2.70062)

Appendix S3. Pairwise F_{ST} differences among all the sampling sites of three subspecies of *Agave sobria* and *A. cerulata* spp. *subcerulata* from the BCP, Mexico. Colors represent F_{ST} values from the lowest of 0 in white to the highest of 0.125 in orange. Sampling sites were arranged according to the subspecies and from the southernmost to the northernmost site. Sampling sites were coded as follows AC - *A. cerulata* ssp. *subcerulata*, ASS - *A. sobria* ssp. *sobria*, ASR - *A. sobria* ssp. *roseana,* ASF - *A. sobria* ssp. *frailensis.*

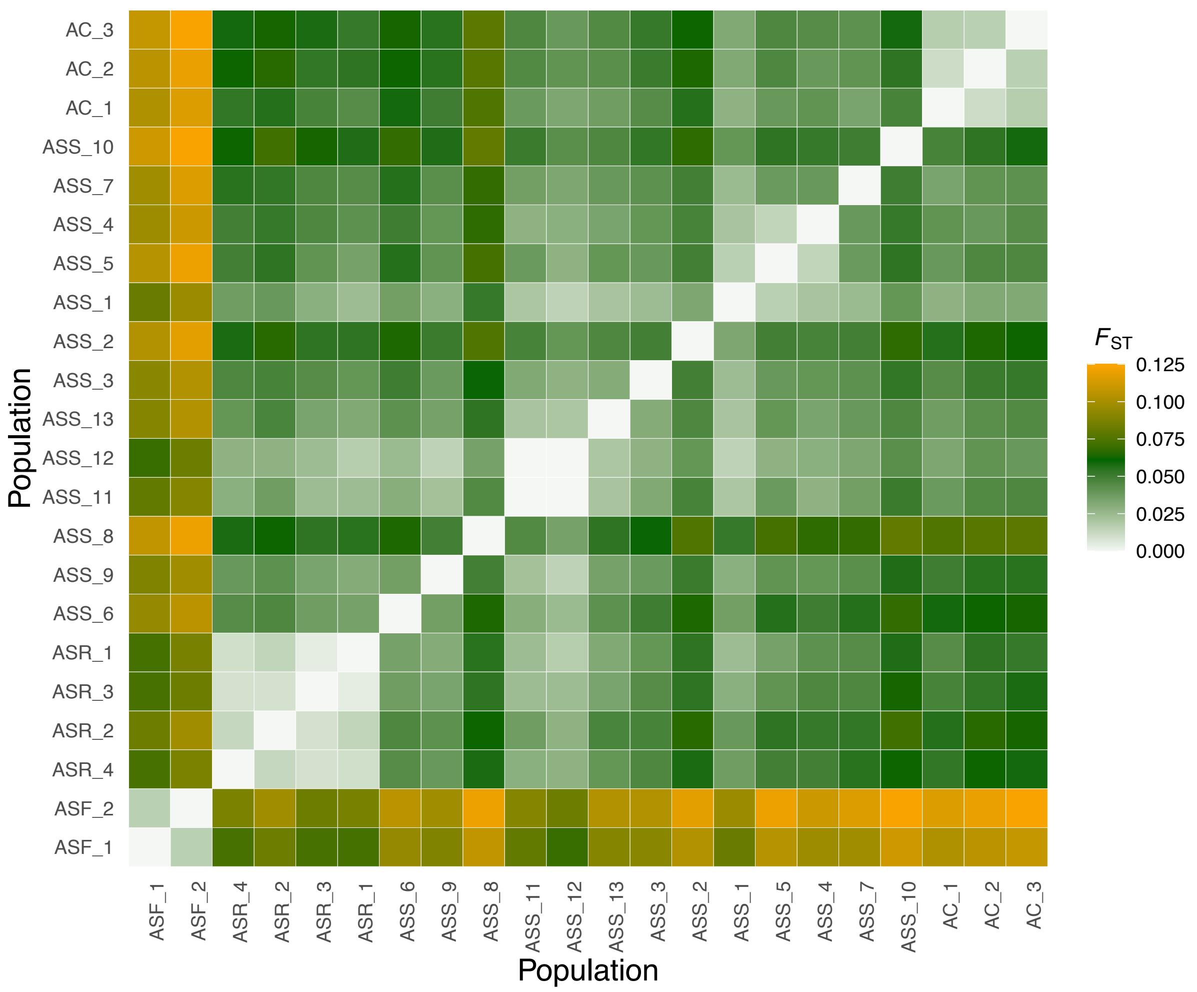

Supplement: Supplementary file 3 — Appendix S3. Pairwise FST differences among all the sampling sites of three subspecies of Agave sobria and A. cerulata spp. subcerulata from the BCP. [file AJB2-112-e70062-s002.pdf]
