# Supplementary material for: The interplay among space, environment, and gene flow drives genetic differentiation in endemic Baja California Agave sobria subspecies
Source: Am J Bot. 2025 Jul 2;112(7):e70062. doi: 10.1002/ajb2.70062 (PMC12281270; doi:10.1002/ajb2.70062)

Appendix S4. Individual-based NJ phylogenetic network of *A. sobria* subspecies and *A. cerulata subcerulata*. NJ tree tips are colored according to the subspecies: dark blue – *A. cerulata* ssp. *subcerulata*, brown – *A. sobria* ssp. *sobria*, light brown – *A. sobria* ssp. *roseana,* and dark cyan - *A. sobria* ssp. *frailensis*.

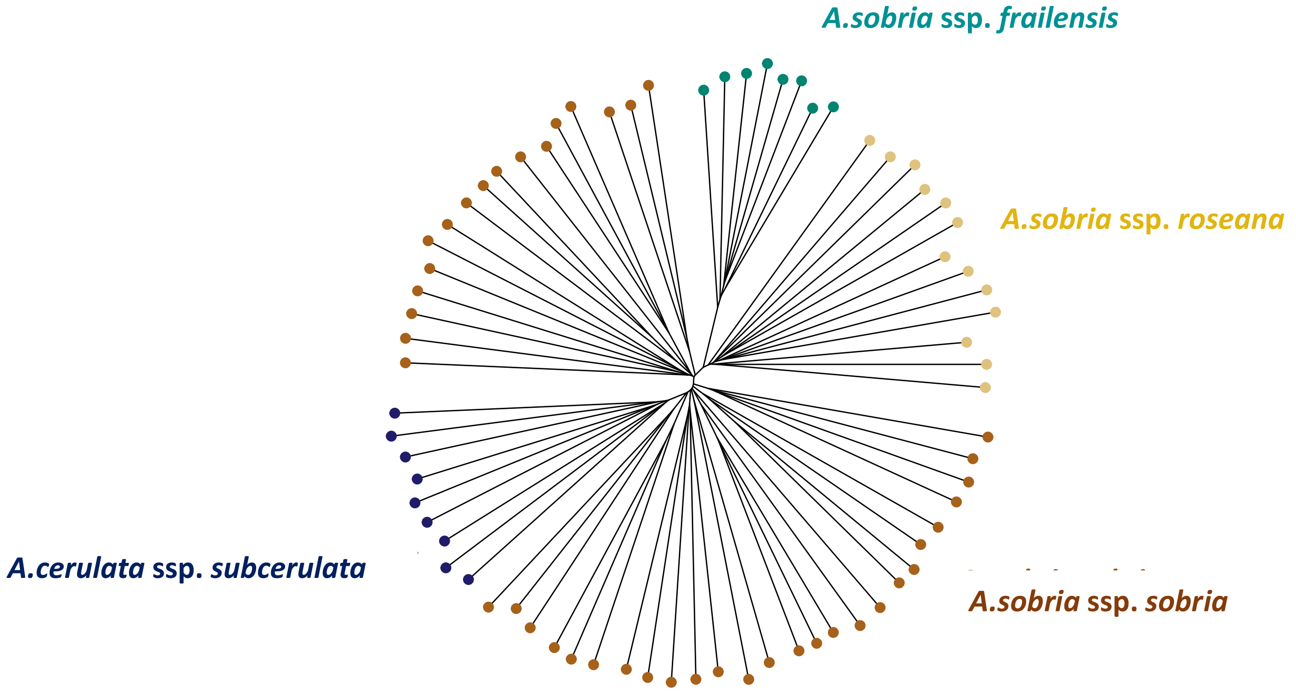

Supplement: Supplementary file 4 — Appendix S4. Individual‐based NJ phylogenetic network of A. sobria subspecies and A. cerulata subcerulata. [file AJB2-112-e70062-s005.pdf]
