# Supplementary material for: The interplay among space, environment, and gene flow drives genetic differentiation in endemic Baja California Agave sobria subspecies
Source: Am J Bot. 2025 Jul 2;112(7):e70062. doi: 10.1002/ajb2.70062 (PMC12281270; doi:10.1002/ajb2.70062)

Appendix S5. A plot of ADMIXTURE cross-validation error and respective standard deviation based on 20 repetitions for each *K* value, from *K*=1 through *K*=10, based on all samples of *A. sobria* and *A. cerulata* and 8453 SNPs.

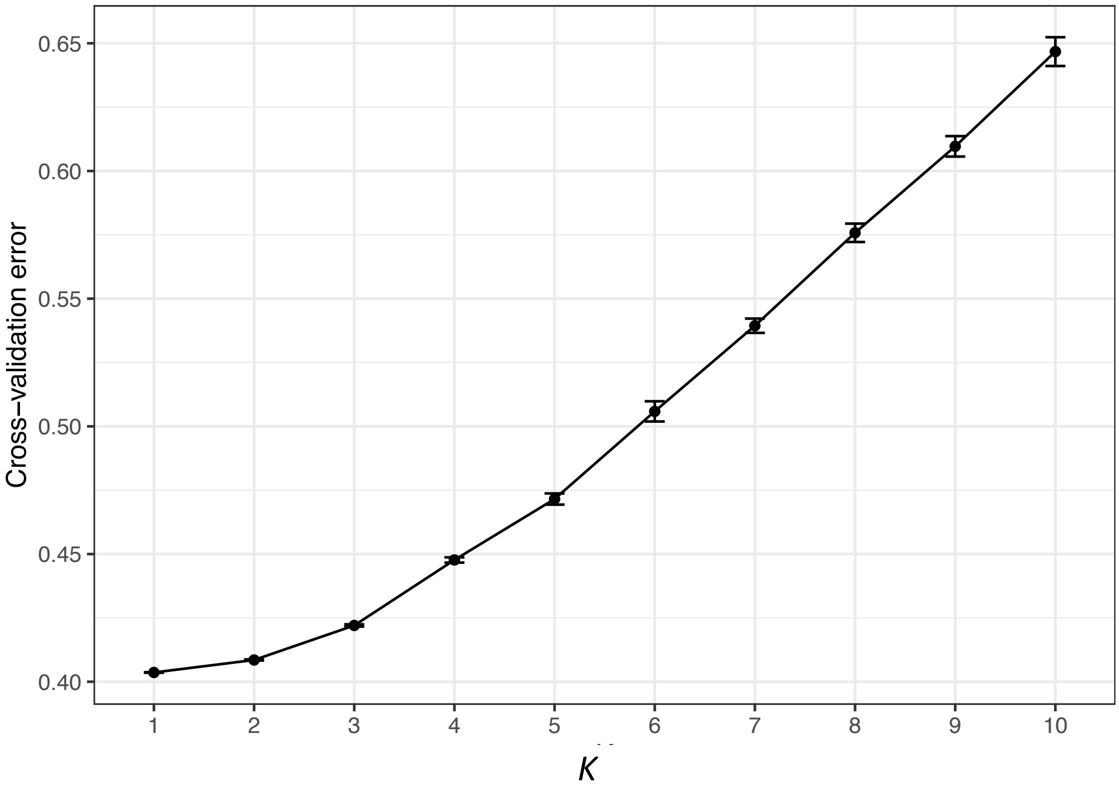

Supplement: Supplementary file 5 — Appendix S5. A plot of ADMIXTURE cross‐validation error and respective standard deviation based on 20 repetitions for each K value, from K = 1 through K = 10, based on all samples of A. sobria and A. cerulata and 8453 SNPs. [file AJB2-112-e70062-s003.pdf]
