# Supplementary material for: The interplay among space, environment, and gene flow drives genetic differentiation in endemic Baja California Agave sobria subspecies
Source: Am J Bot. 2025 Jul 2;112(7):e70062. doi: 10.1002/ajb2.70062 (PMC12281270; doi:10.1002/ajb2.70062)

Appendix S6. Population genetic structure of the *A. sobria* and *A. cerulata* ssp. *subcerulata* samples collected in BCP, Mexico, based on 8453 SNPs. Bar plot of the individual assignment probabilities (vertical axis) for the number of genetic clusters from $K = 2$ to $K = 5$ inferred using the program ADMIXTURE. Samples were arranged according to the subspecies they belonged to, from the southernmost to the northernmost site. The sampling sites were abbreviated as follows: ASF - *A. sobria* ssp. *frailensis*, ASR - *A. sobria* ssp. *roseana*, ASS - *A. sobria* ssp. *sobria* and AC - *A. cerulata* ssp. *subcerulata*. The abbreviations can also be found in Appendix S1.

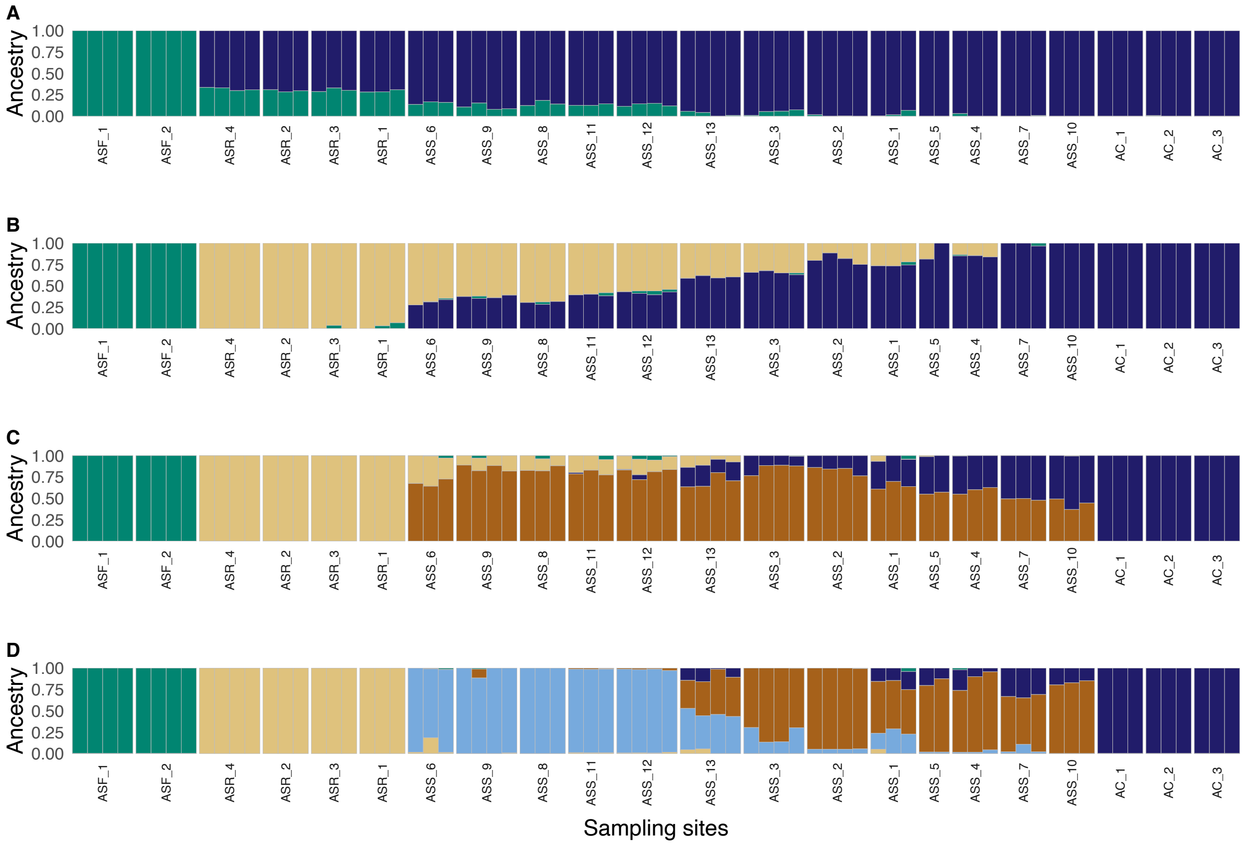

Supplement: Supplementary file 6 — Appendix S6. Population genetic structure of the A. sobria and A. cerulata ssp. subcerulata samples collected on the BCP, based on 8453 SNPs. [file AJB2-112-e70062-s010.pdf]
