# Supplementary material for: The interplay among space, environment, and gene flow drives genetic differentiation in endemic Baja California Agave sobria subspecies
Source: Am J Bot. 2025 Jul 2;112(7):e70062. doi: 10.1002/ajb2.70062 (PMC12281270; doi:10.1002/ajb2.70062)
Supplement: Supplementary file 7 — Appendix S7. Gene flow tests between A. sobria and A. cerulata ssp. subcerulata regions/populations. [file AJB2-112-e70062-s013.pdf]

Appendix S7. Gene flow tests between *A. sobria* and *A. cerulata* ssp. *subcerulata* regions/populations. Tests included all possible quartets considering the topology (((P1, P2), P3), Outgroup). The abbreviations correspond to major geographical regions found with PCA (Figure 3): AC – *A. cerulata* ssp. *subcerulata*, ASF – *A. sobria* ssp. *frailensis,* ASR – *A. sobria* ssp. *roseana,* ASS_N – *A. sobria* ssp *sobria* northern population, ASS_S – *A. sobria* ssp. *sobria* southern population.

P1	P2	P3	Dstatistic	Z-score	*p*-value	f4-ratio	BBAA	ABBA	BABA
AC	ASS_N	ASS_S	0.01948	3.90881	9.28E-05	0.257225	271.944	265.68	255.527
ASF	ASR	ASS_S	0.0272062	3.68094	0.00023238	0.318178	269.791	268.02	253.823
ASF	ASR	AC	0.0260822	3.42028	0.00062557	0.129932	269.857	258.772	245.616
AC	ASS_N	ASR	0.0165894	3.38728	0.0007059	0.121306	270.248	262.784	254.208
ASF	ASR	ASS_N	0.0273506	3.36559	0.00076379	0.298145	268.029	265.52	251.383
AC	ASS_N	ASF	0.0152469	2.87029	0.004101	0.0462121	274.45	252.849	245.255
AC	ASS_S	ASF	0.0161628	2.53042	0.0113927	0.0503345	264.539	260.032	251.76
ASS_N	ASS_S	ASR	0.00138323	0.392609	0.694608	0.0118682	267.331	266.869	266.131
ASS_N	ASS_S	ASF	0.00131777	0.359449	0.719259	0.0043297	272.055	257.395	256.717
ASS_S	ASR	AC	0.00072365	0.12198	0.902915	0.00422415	269.947	260.633	260.257
