# Supplementary material for: The interplay among space, environment, and gene flow drives genetic differentiation in endemic Baja California Agave sobria subspecies
Source: Am J Bot. 2025 Jul 2;112(7):e70062. doi: 10.1002/ajb2.70062 (PMC12281270; doi:10.1002/ajb2.70062)
Supplement: Supplementary file 8 — Appendix S8. The relatedness estimates for within and between subspecies of samples belonging to A. sobria and A. cerulata ssp. subcerulata subspecies collected on the BCP. [file AJB2-112-e70062-s004.pdf]

Appendix S8. The relatedness estimates for within and between subspecies of samples belonging to *A. sobria* and *A. cerulata* ssp. *subcerulata* subspecies collected on the Baja California Peninsula, Mexico. The standard deviation is presented in parentheses.

Subspecies	Relatedness (SD)
A.sobria ssp. *frailensis vs. A.sobria* ssp. *frailensis*	0.17 (0.03)
A.sobria ssp. *frailensis vs.* other subspecies	0.09 (0.024)
A.cerulata ssp. s*ubcerulata* vs. *A. cerulata* ssp. *subcerulata*	0.17 (0.001)
A. cerulata vs. other subspecies	0.13 (0.025)
A.sobria ssp.*sobria* vs. *A. sobria* ssp. *sobria*	0.15 (0.01)
A. sobria ssp*. sobria* vs. other subspecies	0.12 (0.02)
A.sobria ssp*. roseana* vs. *A.sobria* ssp.*roseana*	0.15 (0.02)
A.sobria ssp*. roseana* vs. other subspecies	0.12 (0.02)
