# Supplementary material for: The interplay among space, environment, and gene flow drives genetic differentiation in endemic Baja California Agave sobria subspecies
Source: Am J Bot. 2025 Jul 2;112(7):e70062. doi: 10.1002/ajb2.70062 (PMC12281270; doi:10.1002/ajb2.70062)

Appendix S9. Mantel test results show the relationships between geographic distance and genetic distance as estimates with pairwise F_{ST} among sampling sites of *A. sobria* and *A. cerulata* ssp. s*ubcerulata* from the Baja California Peninsula.

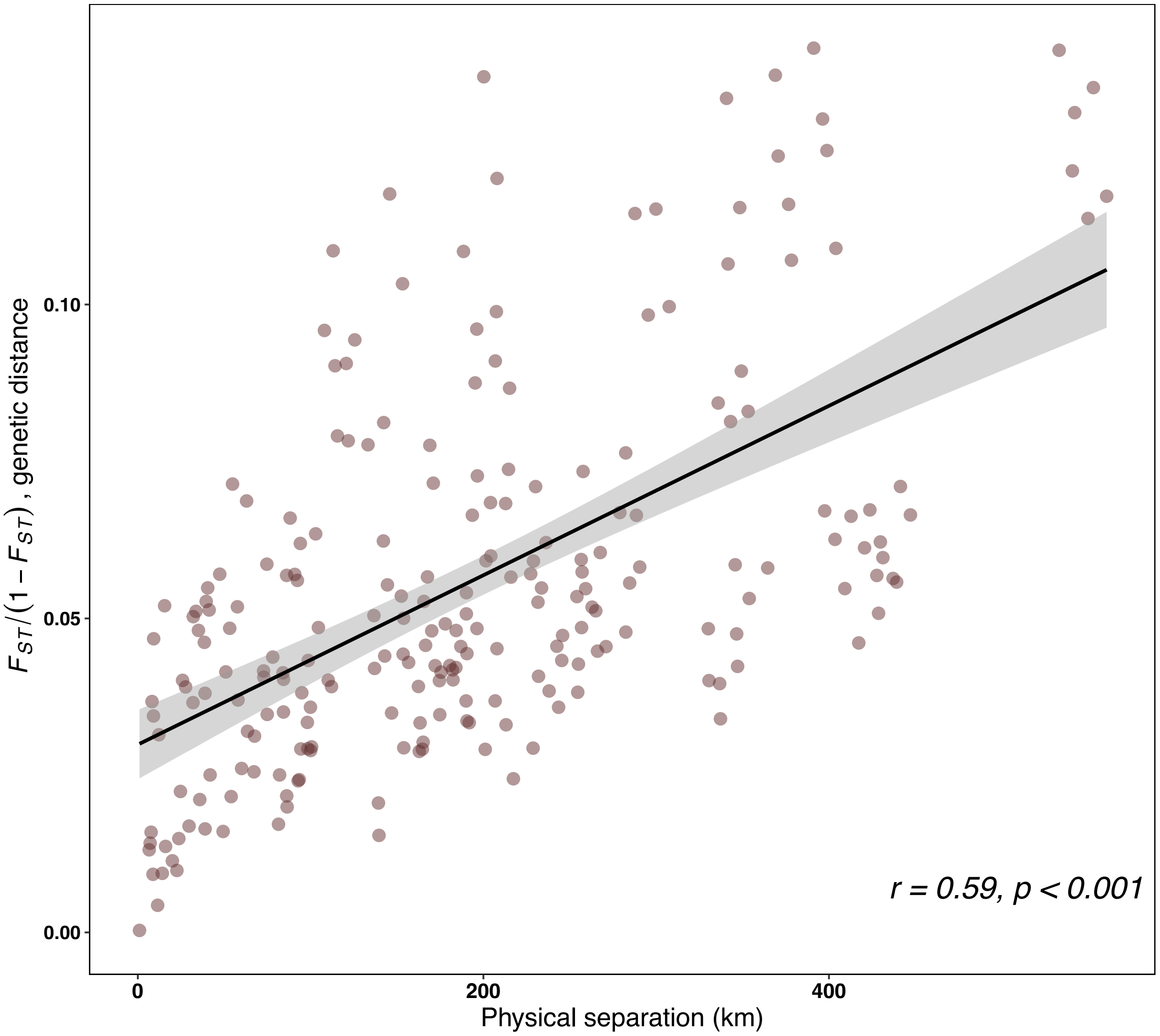

Supplement: Supplementary file 9 — Appendix S9. Mantel test results show the relationships between geographic distance and genetic distance as estimates with pairwise F ST among sampling sites of A. sobria and A. cerulata ssp. subcerulata from the BCP. [file AJB2-112-e70062-s011.pdf]
