# Supplementary material for: The interplay among space, environment, and gene flow drives genetic differentiation in endemic Baja California Agave sobria subspecies
Source: Am J Bot. 2025 Jul 2;112(7):e70062. doi: 10.1002/ajb2.70062 (PMC12281270; doi:10.1002/ajb2.70062)
Supplement: Supplementary file 10 — Appendix S10. Mantel and partial Mantel tests summarizing relationships (r and associated p values) between genetic distance, geographic distance, and climate variables in A. sobria. [file AJB2-112-e70062-s012.pdf]

Appendix S10. Mantel and partial Mantel tests summarizing relationships (*r* and associated *p* values) between genetic distance, geographic distance, and climate variables in *A. sobria, P-values*p<0.05; **p<0.01; ***p<0.001*

	Mantel, r	Partial Mantel, r
Geographic distance	0.59***	NA
Annual Mean Temperature (BIO1)	0.18*	-0.07
Mean diurnal range (BIO 2)	0.65**	**0.56****
Isothermality (BIO 3)	0.38**	**0.39****
Temperature Seasonality (BIO 4)	0.07	-0.05
Max Temperature of Warmest Month (BIO5)	0.48***	**0.37****
Min Temperature of Coldest Month (BIO6)	0.49***	**0.25***
Temperature Annual Range (BIO7)	0.65***	**0.45****
Mean Temperature of Wettest Quarter (BIO8)	0.08	0.14
Mean Temperature of Driest Quarter (BIO9)	0.07	-0.05
Mean Temperature of Warmest Quarter (BIO 10)	0.09	0.05
Mean Temperature of Coldest Quarter (BIO11)	0.18*	-0.07
Annual Precipitation (BIO12)	0.34**	0.08
Precipitation of Wettest Month (BIO13)	0.58***	**0.33****
Precipitation of Driest Month (BIO 14)	-0.04	-0.14
Precipitation Seasonality (BIO 15)	0.29*	-0.22
Precipitation of Wettest Quarter (BIO 16)	0.38**	0.06
Precipitation of Driest Quarter (BIO 17)	0.02	0.09
Precipitation of Warmest Quarter (BIO 18)	0.26*	-0.08
Precipitation of Coldest Quarter (BIO 19)	0.09	-0.02
