# Supplementary material for: The interplay among space, environment, and gene flow drives genetic differentiation in endemic Baja California Agave sobria subspecies
Source: Am J Bot. 2025 Jul 2;112(7):e70062. doi: 10.1002/ajb2.70062 (PMC12281270; doi:10.1002/ajb2.70062)
Supplement: Supplementary file 11 — Appendix S11. Results of BLASTx analysis of transcripts containing putative outlier loci. [file AJB2-112-e70062-s001.pdf]

Appendix S11. Results of BLASTx analysis of transcripts containing putative outlier loci.

Transcript ID	% of identity	e-value	Protein Name	Organism
GAHU01003755.1	96.86	6.00E-97	NADH dehydrogenase [ubiquinone] iron-sulfur protein 7, mitochondrial-like	Nicotiana tabacum (Common tobacco)
GAHU01003980.1	89.91	2.00E-57	S5 DRBM domain-containing protein	Cannabis sativa (Hemp) (Marijuana)
GAHU01004031.1	93.79	0	transketolase (EC 2.2.1.1)	Asparagus officinalis (Garden asparagus)
GAHU01005974.1	39.39	7.00E-17	Zinc finger CCHC-type superfamily	Arabidopsis suecica (Swedish thale-cress) (Cardaminopsis suecica)
GAHU01006267.1	97.98	7.00E-115	Ribosomal protein L15	Asparagus officinalis (Garden asparagus)
GAHU01009161.1	77.7	7.00E-157	RING-type domain-containing protein	Asparagus officinalis (Garden asparagus)
GAHU01009464.1	88.38	3.00E-175	Hypersensitive-induced response protein 4 isoform X1	Elaeis guineensis var. tenera (Oil palm)
GAHU01009886.1	70.12	4.00E-150	AIG1-type G domain-containing protein	Asparagus officinalis (Garden asparagus)
GAHU01010663.1	79.1	6.00E-125	ER membrane protein complex subunit 10	Dioscorea zingiberensis
GAHU01011049.1	65.56	3.00E-45	DUF1645 domain-containing protein	Asparagus officinalis (Garden asparagus)
GAHU01012150.1	72.44	1.00E-108	BHLH transcription factor	Dracaena cambodiana
GAHU01012587.1	81.82	4.00E-36	poly(A)-specific ribonuclease (EC 3.1.13.4)	Asparagus officinalis (Garden asparagus)
GAHU01012732.1	90.59	0	Calcium-transporting ATPase (EC 7.2.2.10)	Asparagus officinalis (Garden asparagus)
GAHU01014918.1	77.78	0	Zinc finger CCCH domain-containing protein ZFN-like isoform X1	Elaeis guineensis var. tenera (Oil palm)
GAHU01015907.1	91.5	2.00E-115	Arf-GAP domain-containing protein	Asparagus officinalis (Garden asparagus)
GAHU01016563.1	81.34	6.00E-110	UBC core domain-containing protein	Asparagus officinalis (Garden asparagus)
GAHU01017311.1	97.96	0	Cytochrome b	Capsella bursa-pastoris (Shepherd's purse) (Thlaspi bursa-pastoris)
GAHU01018072.1	81.79	0	Dihydroflavonol 4-reductase	Phoenix dactylifera (Date palm)
GAHU01018250.1	95.52	1.00E-30	LOW QUALITY PROTEIN: oxysterol-binding protein-related protein 3C	Phoenix dactylifera (Date palm)
GAHU01019358.1	80.82	0	Cellulose synthase-like protein D2	Elaeis guineensis var. tenera (Oil palm)
GAHU01019537.1	84.77	0	DJ-1/PfpI domain-containing protein	Asparagus officinalis (Garden asparagus)
GAHU01020701.1	79.52	0	Inosine-5'-monophosphate dehydrogenase (IMP dehydrogenase) (IMPD) (IMPDH) (EC 1.1.1.205)	Elaeis guineensis var. tenera (Oil palm)
GAHU01020972.1	86.26	0	phosphoribosylformylglycinamidine synthase (EC 6.3.5.3) (Formylglycinamide ribonucleotide amidotransferase) (Formylglycinamide ribotide amidotransferase)	Asparagus officinalis (Garden asparagus)

GAHU01021001.1	92.31	2.00E-135	Uncharacterized protein	Asparagus officinalis (Garden asparagus)
GAHU01021221.1	69.11	3.00E-124	Transmembrane protein	Asparagus officinalis (Garden asparagus)
GAHU01022712.1	93.09	2.00E-144	RHOMBOID-like protein (EC 3.4.21.105)	Yucca filamentosa (Bear-grass) (Adam's-needle)
GAHU01023782.1	53.28	2.00E-109	Uncharacterized protein	Asparagus officinalis (Garden asparagus)
GAHU01024011.1	63.33	4.00E-113	AB hydrolase-1 domain-containing protein	Asparagus officinalis (Garden asparagus)
GAHU01024822.1	91.72	5.00E-96	EF-hand domain-containing protein	Asparagus officinalis (Garden asparagus)
GAHU01026001.1	76.66	0	WEB family protein	Asparagus officinalis (Garden asparagus)
GAHU01026627.1	80.73	0	Mediator of RNA polymerase II transcription subunit 14 (Mediator complex subunit 14)	Asparagus officinalis (Garden asparagus)
GAHU01027787.1	71.55	9.00E-39	Triose-phosphate Transporter family	Musa troglodytarum
GAHU01028666.1	87.56	0	Thioredoxin domain-containing protein	Asparagus officinalis (Garden asparagus)
GAHU01029060.1	84.34	0	RCK N-terminal domain-containing protein	Asparagus officinalis (Garden asparagus)
GAHU01029757.1	54.78	3.00E-13	PREDICTED: EUGRSUZ_I01330	Prunus dulcis (Almond) (Amygdalus dulcis)
GAHU01030702.1	62.72	3.00E-115	BHLH transcription factor	Dracaena cambodiana
GAHU01032051.1	84.38	0	Phytochrome B	Cocos nucifera (Coconut palm)
GAHU01032179.1	75.05	0		
GAHU01032711.1	96.4	4.00E-53	Histone H2A	Elaeis guineensis var. tenera (Oil palm)
GAHU01035874.1	72.68	9.00E-61	protein-serine/threonine phosphatase (EC 3.1.3.16)	Asparagus officinalis (Garden asparagus)
GAHU01036447.1	62.65	4.00E-60	Remorin 4.1-like	Phoenix dactylifera (Date palm)
GAHU01036735.1	46.9	1.00E-75	(wild Malaysian banana) hypothetical protein	Musa acuminata subsp. malaccensis (Wild banana) (Musa malaccensis)
GAHU01036787.1	71.68	3.00E-140	Thaumatin-like protein 1	Dioscorea zingiberensis
GAHU01036894.1	61.88	3.00E-152	Mitochondrial inner membrane protein OXA1-like	Musa troglodytarum
GAHU01041028.1	58.24	1.00E-56	DUF4228 domain-containing protein	Ensete ventricosum (Abyssinian banana) (Musa ensete)
GAHU01041160.1	66.38	6.00E-138	(wild Malaysian banana) hypothetical protein	Musa acuminata subsp. malaccensis (Wild banana) (Musa malaccensis)
GAHU01041213.1	62.99	0	RNA helicase (EC 3.6.4.13)	Dioscorea zingiberensis
GAHU01042143.1	76.17	3.00E-113	Survival of motor neuron-related-splicing factor 30 (Survival motor neuron domain-containing protein 1)	Elaeis guineensis var. tenera (Oil palm)
GAHU01042301.1	60.39	0	Uncharacterized protein LOC105044594 isoform X1	Elaeis guineensis var. tenera (Oil palm)
GAHU01043280.1	79.19	1.00E-102	CLK4-associating serine/arginine rich protein isoform X5	Phoenix dactylifera (Date palm)

GAHU01044146.1	70.87	8.00E-109	Myb-related protein Hv1	Carex littledalei
GAHU01044815.1	95.69	2.00E-67	Glycerol-3-phosphate dehydrogenase [NAD(+)] (EC 1.1.1.8)	Asparagus officinalis (Garden asparagus)
GAHU01045166.1	82.47	2.00E-59	RRM domain-containing protein	Asparagus officinalis (Garden asparagus)
GAHU01045228.1	55.89	2.00E-166	Premnaspirodiene oxygenase-like	Phoenix dactylifera (Date palm)
GAHU01045555.1	61.16	0	DUF936 domain-containing protein	Asparagus officinalis (Garden asparagus)
GAHU01046427.1	74.61	0	Protein kinase domain-containing protein	Asparagus officinalis (Garden asparagus)
GAHU01047135.1	67.27	0	CW-type domain-containing protein	Asparagus officinalis (Garden asparagus)
GAHU01047139.1	79.51	0	histidine kinase (EC 2.7.13.3)	Elaeis guineensis var. tenera (Oil palm)
GAHU01047271.1	45.5	3.00E-121	RPM1 interacting protein 13	Asparagus officinalis (Garden asparagus)
GAHU01047280.1	89.18	0	tryptophan synthase (EC 4.2.1.20)	Asparagus officinalis (Garden asparagus)
GAHU01047742.1	52.32	1.00E-129	Zinc finger, C3HC4 type (RING finger)	Musa troglodytarum
GAHU01051757.1	74.1	0	UPF0481 protein	Musa troglodytarum
GAHU01052605.1	48.3	4.00E-94	RING-type E3 ubiquitin transferase (EC 2.3.2.27)	Apostasia shenzhenica
GAHU01052947.1	71.18	0	Receptor protein kinase-like protein ZAR1	Phoenix dactylifera (Date palm)
GAHU01057487.1	59.68	0	RING-type E3 ubiquitin transferase (EC 2.3.2.27)	Elaeis guineensis var. tenera (Oil palm)
GAHU01059580.1	82.89	3.00E-51	protein-serine/threonine phosphatase (EC 3.1.3.16)	Asparagus officinalis (Garden asparagus)
GAHU01061275.1	86.53	0	Exportin-1/Importin-beta-like domain-containing protein	Asparagus officinalis (Garden asparagus)
GAHU01062355.1	48.7	7.00E-45	Uncharacterized protein	Dioscorea zingiberensis
GAHU01063346.1	66.19	0	LRR receptor-like serine threonine-protein kinase	Musa troglodytarum
GAHU01067854.1	65.69	2.00E-99	MYB-like transcription factor ODO1	Phoenix dactylifera (Date palm)
GAHU01067950.1	60.9	1.00E-34	Uncharacterized protein	Dioscorea zingiberensis
GAHU01068612.1	84.38	0	C2 and GRAM domain-containing protein	Asparagus officinalis (Garden asparagus)
GAHU01068649.1	69.12	3.00E-78	RING-CH-type domain-containing protein	Dendrobium nobile (Orchid)
GAHU01070088.1	83.81	0	ABC transporter domain-containing protein	Asparagus officinalis (Garden asparagus)
GAHU01070984.1	79.19	0	Enhancer of polycomb-like protein	Asparagus officinalis (Garden asparagus)
GAHU01072217.1	66.42	0	Rab-GAP TBC domain-containing protein	Dendrobium catenatum
GAHU01073802.1	85.01	0	Uncharacterized protein	Asparagus officinalis (Garden asparagus)
GAHU01076940.1	68.82	0	Pectinesterase (EC 3.1.1.11)	Phoenix dactylifera (Date palm)
GAHU01080077.1	56.65	7.00E-64	LOB domain-containing protein	Colocasia esculenta (Wild taro) (Arum esculentum)
GAHU01081445.1	64.13	2.00E-99	AP2/ERF domain-containing protein	Asparagus officinalis (Garden asparagus)
GAHU01081720.1	81.41	0	1-acylglycerol-3-phosphate O-acyltransferase (EC 2.3.1.51)	Asparagus officinalis (Garden asparagus)

GAHU01081973.1	86.11	6.00E-24	Subtilisin-like protease SDD1	Anthurium amnicola
GAHU01084894.1	58.89	2.00E-17	Uncharacterized protein	Asparagus officinalis (Garden asparagus)
GAHU01087298.1	92.77	6.00E-44	ARF guanine-nucleotide exchange factor GNOM-like	Asparagus officinalis (Garden asparagus)
GAHU01088149.1	83.78	9.00E-12	Uncharacterized protein	Asparagus officinalis (Garden asparagus)
GAHU01088249.1	61.82	3.00E-122	Transcription factor MYB36-like	Elaeis guineensis var. tenera (Oil palm)
GAHU01093663.1	70.07	2.00E-55	RNA uridylyltransferase (EC 2.7.7.52)	Asparagus officinalis (Garden asparagus)
GAHU01094026.1	78.04	0	non-specific serine/threonine protein kinase (EC 2.7.11.1)	Asparagus officinalis (Garden asparagus)
GAHU01094942.1	75.83	1.00E-51	Ribosome assembly factor mrt4	Elaeis guineensis var. tenera (Oil palm)
GAHU01097999.1	80.77	8.00E-21	CULT domain-containing protein	Dendrobium catenatum
GAHU01100824.1	76.42	0	Purple acid phosphatase (EC 3.1.3.2)	Musa acuminata subsp. malaccensis (Wild banana) (Musa malaccensis)
GAHU01102179.1	70.07	0	Protein PHOX1	Phoenix dactylifera (Date palm)
GAHU01102893.1	72.36	0	Probably inactive leucine-rich repeat receptor-like protein kinase IMK2	Elaeis guineensis var. tenera (Oil palm)
GAHU01104284.1	72.73	4.00E-106	NDR1/HIN1-like protein 6	Phoenix dactylifera (Date palm)
GAHU01114700.1	52.11	2.00E-13	Protein kinase domain-containing protein	Asparagus officinalis (Garden asparagus)
GAHU01121627.1	85.81	4.00E-85	Protein terminal ear1-like	Phoenix dactylifera (Date palm)
GAHU01125963.1	72.13	0	DNA endonuclease activator Ctp1 C-terminal domain-containing protein	Asparagus officinalis (Garden asparagus)
GAHU01132561.1	68.81	0	DYW domain-containing protein	Asparagus officinalis (Garden asparagus)
GAHU01149996.1	40.83	8.00E-20	Pentatricopeptide repeat-containing protein At1g05670, mitochondrial-like isoform X2	Phoenix dactylifera (Date palm)
GAHU01161811.1	73.46	0	3-ketoacyl-CoA synthase (EC 2.3.1.-)	Artemisia annua (Sweet wormwood)
GAHU01174864.1	40.55	2.00E-50	RING-type E3 ubiquitin transferase (EC 2.3.2.27)	Dioscorea zingiberensis
GAHU01186905.1	84.5	6.00E-158	Flavin-containing monooxygenase (EC 1.-.-.-)	Asparagus officinalis (Garden asparagus)
