# Supplementary material for: The interplay among space, environment, and gene flow drives genetic differentiation in endemic Baja California Agave sobria subspecies
Source: Am J Bot. 2025 Jul 2;112(7):e70062. doi: 10.1002/ajb2.70062 (PMC12281270; doi:10.1002/ajb2.70062)
Supplement: Supplementary file 12 — Appendix S12. List of significantly enriched gene ontology (GO) terms for transcripts of A. sobria containing outlier markers. [file AJB2-112-e70062-s007.pdf]

Appendix S12. List of significantly enriched gene ontology (GO) terms for transcripts of *A. sobria* containing outlier markers. Biological processes associated with responses to environmental factors are shown in bold.

GO Term ID	Ontology	Function	*p*-value	Adjusted *p*-value
GO:0009060	BP	aerobic respiration	0.002	0.030
**GO:0007276**	**BP**	**gamete generation**	**0.005**	**0.030**
GO:0061157	BP	mRNA destabilization	0.006	0.030
GO:0006402	BP	mRNA catabolic process	0.008	0.030
GO:0010196	BP	nonphotochemical quenching	0.009	0.030
GO:0006177	BP	GMP biosynthetic process	0.012	0.030
GO:0022904	BP	respiratory electron transport chain	0.013	0.030
**GO:0010212**	**BP**	**response to ionizing radiation**	**0.014**	**0.030**
GO:1900364	BP	negative regulation of mRNA polyadenylation	0.015	0.030
GO:0015990	BP	electron transport coupled proton transport	0.015	0.030
GO:0046168	BP	glycerol-3-phosphate catabolic process	0.017	0.030
**GO:1905421**	**BP**	**regulation of plant organ morphogenesis**	**0.021**	**0.036**
**GO:0009631**	**BP**	**cold acclimation**	**0.034**	**0.044**
GO:0032981	BP	mitochondrial respiratory chain complex I assembly	0.034	0.044
**GO:0009584**	**BP**	**detection of visible light**	**0.036**	**0.044**
GO:0045036	BP	protein targeting to chloroplast	0.037	0.044
GO:0009627	BP	systemic acquired resistance	0.037	0.044
GO:0008284	BP	positive regulation of cell population proliferation	0.041	0.044
GO:0009094	BP	L-phenylalanine biosynthetic process	0.042	0.044
GO:0045275	CC	respiratory chain complex III	0.004	0.030
GO:0005747	CC	mitochondrial respiratory chain complex I	0.023	0.037
GO:0042651	CC	thylakoid membrane	0.026	0.041
GO:0009331	CC	glycerol-3-phosphate dehydrogenase complex	0.032	0.044
GO:0019172	MF	glyoxalase III activity	0.007	0.030
GO:0004535	MF	poly(A)-specific ribonuclease activity	0.011	0.030
GO:0000155	MF	phosphorelay sensor kinase activity	0.012	0.030
GO:0003938	MF	IMP dehydrogenase activity	0.013	0.030
GO:0103075	MF	indole-3-pyruvate monooxygenase activity	0.014	0.030
GO:0008121	MF	ubiquinol-cytochrome-c reductase activity	0.014	0.030
GO:0047952	MF	glycerol-3-phosphate dehydrogenase [NAD(P)+] activity	0.015	0.030
GO:0008143	MF	poly(A) binding	0.016	0.030
GO:0032574	MF	5'-3' RNA helicase activity	0.016	0.030
GO:0003841	MF	1-acylglycerol-3-phosphate O-acyltransferase activity	0.017	0.030
GO:0004802	MF	transketolase activity	0.027	0.041
GO:0032977	MF	membrane insertase activity	0.037	0.044
GO:0009922	MF	fatty acid elongase activity	0.040	0.044

GO:0004664	MF	prephenate dehydratase activity	0.042	0.044
GO:0004499	MF	N,N-dimethylaniline monooxygenase activity	0.043	0.044
GO:0004642	MF	phosphoribosylformylglycinamidine synthase activity	0.048	0.048
