# Supplementary material for: The interplay among space, environment, and gene flow drives genetic differentiation in endemic Baja California Agave sobria subspecies
Source: Am J Bot. 2025 Jul 2;112(7):e70062. doi: 10.1002/ajb2.70062 (PMC12281270; doi:10.1002/ajb2.70062)

Appendix S13. NJ phylogenetic networks of individuals of *A. sobria* based on 110 random SNPs. NJ tree tips are colored according to the populations identified with PCA, brown – northern *A. sobria* ssp. *sobria*, blue – southern *A. sobria* ssp. *sobria*, light brown – *A. sobria* ssp. *roseana*, dark cyan - *A. sobria* ssp. *frailensis*.

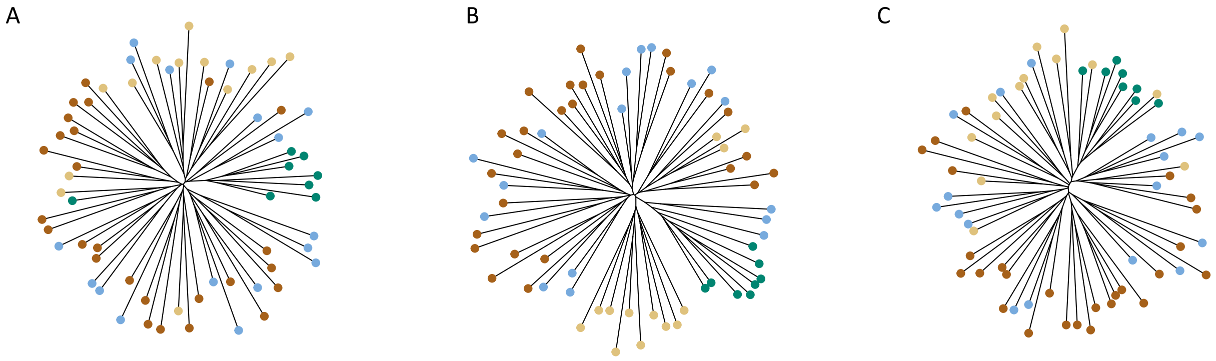

Supplement: Supplementary file 13 — Appendix S13. NJ phylogenetic networks of individuals of A. sobria based on 110 random SNPs. [file AJB2-112-e70062-s006.pdf]
